# Integrative analysis of anti-breast CancerPotential of metabolites from *Pseudomonas frederiksbergensis* isolated from Taoerqi

**DOI:** 10.3389/fphar.2025.1469949

**Published:** 2025-03-28

**Authors:** Yuexing Ma, Haoyi Zheng, Simin Liu, En Yuan, Xin Qiao, Zhang Dai, Wenli Wu, Rongbin Pan

**Affiliations:** ^1^ College of Chinese Medicine, China Pharmaceutical University, Nanjing, Jiangsu, China; ^2^ Jiangxi University of Chinese Medicine, Nanchang, Jiangxi, China; ^3^ Science and Technology College, Jiangxi University of Chinese Medicine, Nanchang, Jiangxi, China; ^4^ Pharmacy Colledge, Key Laboratory of Key Technology and Application of Drug Screening for Inflammatory Diseases and Inflammation, Jiangxi Provincial Department of Education, Nanchang Medical College, Nanchang, Jiangxi, China; ^5^ School of Biomedical Sciences, University of Queensland, Brisbane, QLD, Australia; ^6^ Jiangzhong Cancer Research Center, Jiangxi University of Chinese Medicine, Nanchang, Jiangxi, China

**Keywords:** Taoerqi, *Pseudomonas frederiksbergensis*, endophytic metabolites, breast cancer, CancerPotential

## Abstract

**Introduction:**

The Tibetan medicinal botanical drug Taoerqi has long been recognized for its anti-inflammatory, antibacterial, and tumor-inhibitory properties.

**Methods:**

Botanical drug focuses on the isolation and characterization of secondary metabolites from *Pseudomonas frederiksbergensis*, an endophytic bacterium isolated from Taoerqi roots. The metabolites were obtained through fermentation and purification processes and were evaluated for their anti-breast cancer activities using cellular assays and transcriptomic analysis. Key regulatory targets, including SARM1, RGS5, PROM2, and BAG1, were identified through bioinformatics analysis and validated using qPCR and Western blotting. Furthermore, a clinical risk assessment model was constructed using breast cancer transcriptome databases to explore the potential prognostic value of these targets.

**Results:**

The secondary metabolites from *Pseudomonas frederiksbergensis* exhibit significant anti-tumor effects and highlight their potential molecular mechanisms in breast cancer regulation.

**Discussion:**

This study provides insights into the therapeutic potential of these metabolites and lays the groundwork for future preclinical and *in vivo* investigations.

## 1 Introduction

*Podophyllum hexandrum* (Royle) Ying [Berberidaceae], Pharmacopoeia of the People’s Republic of China (2020 Edition), commonly referred to as Taoerqi in Tibetan medicine, is a traditional botanical drug widely recognized for its anti-inflammatory, antibacterial, and anticancer properties (Yaohui et al., 2014a; Yaohui et al., 2013). Historically, its roots, rhizomes, and fruits have been used to treat gynecological disorders, regulate menstrual flow, and promote blood circulation. Among these, podophyllotoxin is the primary secondary metabolite, serving as a precursor for clinically used anticancer drugs like etoposide and teniposide (Yaohui et al., 2014b). However, the high toxicity of the roots and rhizomes restricts their direct clinical application, leading to increased interest in alternative sources of bioactive metabolites.

In recent years, endophytic microorganisms isolated from *P. hexandrum* have garnered attention due to their ability to produce pharmacologically active secondary metabolites. Among them, *P. frederiksbergensis*, an endophytic bacteriu, we first isolated from the roots of *P. hexandrum*, has shown promising biological activities, including environmental bioremediation and potential anticancer effects (Ting, 2016) Originally identified from coal gasification sites, *Pseudomonas frederiksbergensis* demonstrates an exceptional capacity to degrade toxic polycyclic aromatic hydrocarbons (PAHs), such as phenanthrene and anthracene (Aislabie et al., 2006). In addition to its ability to degrade these hydrocarbons, *P. frederiksbergensis* has been shown to break down various aromatic compounds, including phthalates and hydroxybenzoic acid, highlighting its promise in environmental remediation (Biocyc Pathway Database, 2023). Beyond its environmental bioremediation capabilities, recent studies have also focused on the bioactive metabolites of *P*. *frederiksbergensis*, which exhibit notable cytotoxicity against various cancer cell lines. These metabolites have been shown to possess anticancer properties by affecting gene expression, modulating cell cycle progression, and inducing apoptosis (Liu et al., 2023; Kumar et al., 2019; Bashizi et al., 2023). Such findings suggest that *P. frederiksbergensis* may offer promising therapeutic strategies in cancer treatment, with significant potential for novel drug development.

Despite growing interest in the biotechnological and pharmaceutical potential of *P*. *frederiksbergensis*, its specific anticancer mechanisms, especially in breast cancer, remain largely unexplored. The goal of this study was to resolve the potential mechanisms of *P*. *frederiksbergensis* metabolites against breast cancer, and to validate the effects of the metabolites on the tumor microenvironment through cellular experiments, transcriptome analysis, and bioinformatics modeling. To address this knowledge gap, our study integrates cellular assays, transcriptomic analysis, and clinical database mining to evaluate the effects of secondary metabolites from *P. frederiksbergensis* on breast cancer cells. This investigation, utilizing a bioinformatics-clinical prognostic model, focuses on key regulatory genes, including SARM1, RGS5, PROM2, and BAG1, which are closely associated with tumor progression, immune regulation, and cellular apoptosis pathways. (Piezzo et al., 2020; Andersen et al., 2000).

Furthermore, bioinformatics analysis was employed to construct a breast cancer risk prognosis model, evaluating the potential clinical relevance of these genes as therapeutic targets. By combining transcriptome data and molecular validation, this study aims to provide novel insights into the anticancer mechanisms of *P. frederiksbergensis* metabolites and establish a foundation for future preclinical investigations.

## 2 Materials and methods

### 2.1 Isolation and purification of endophytic bacteria from plants

#### 2.1.1 Plant material

*Podophyllum hexandrum* Royle [Berberidaceae] (Taoerqi) was collected on 26 September 2020, from Gansu Dingxi (altitude 2614.7 m, longitude 104.545791, latitude 34.508744). Identified as medicinal plant Taoerqi by Teacher Shengfu Hu(Jiangxi University of Chinese Medicine), its well-grown roots were collected and stored in a refrigerator at 4°C for further use.

The medicinal materials used in this study fully comply with the Nagoya Protocol, CITES, and all associated treaties, including phytosanitary regulations.

#### 2.1.2 Preparation of culture media

In this experiment, the isolation of endophytic fungi was carried out using Potato Dextrose Agar (PDA) solid culture medium. PDA solid medium was packed and sterilized by high-pressure steam at 121°C for 20 min. Potato Dextrose Broth (PDB) was used for the liquid culture of endophytic bacteria, with each bottle containing 250 mL.

#### 2.1.3 Methods for isolating and purifying endophytic bacteria

① Isolation of endophytic fungi: Freshly collected roots were washed to remove surface attachments and dried with sterile filter paper. Then, the roots were cut into small segments of 4 cm in length. These segments were subjected to surface sterilization and disinfection under aseptic conditions in a laminar flow hood as follows:

Sterilization procedure: rinsing with sterile water for 30 s → soaking in 75% alcohol for 30 s → soaking in 2% sodium hypochlorite solution for 2 min → soaking in 75% alcohol for 30 s → rinsing three times with sterile water.

The treated roots were then cut in half using a sterile blade and inoculated onto PDA culture medium plates, placed in a constant temperature incubator at 28°C for static culture for 2–7 days. During this period, the growth of endophytic fungi was observed. Once the mycelium grew at the edge of the tissue, a portion was picked under sterile conditions for purification on culture plates. After multiple purifications, the cultures were transferred and stored in test tubes for future use.

② Surface sterilization verification experiments: In the process of isolating endophytic fungi, three types of blank control experiments were set up to ensure that the obtained strains were plant endophytic fungi.

Method one: Three open PDA culture plates were placed in the laminar flow hood during the isolation process. After incubating at 28°C for 7 days, no contaminants should appear on these plates, ensuring that the colonies grown on the isolation plates originated from plant materials rather than environmental contaminants.

Method two: Rinse liquid test, sterile water from the final rinse was spread onto PDA culture plates and incubated at 28°C for 7 days.

Method three: Tissue imprinting method, where the surface-sterilized plant materials were placed on PDA culture plates. After 20 min of contact between the sterilized plant material and PDA culture medium, the sterilized plant material was removed, and the plates were incubated at 28°C for 7 days.

If no colonies appeared on the above PDA culture plates after incubation, it indicated that the surface sterilization of the plant was thorough, and surface bacteria had been completely eliminated, while the colonies growing on the plant incision were endophytic bacteria.

#### 2.1.4 Purification and preservation of endophytic bacteria

The purification of endophytic bacteria mainly used the quadrant streak method, which is employed to isolate and purify bacterial strains. It divides the plate into four quadrants, with the fourth quadrant being the main distribution area of single colonies. Under aseptic conditions, a small amount of bacterial cells was directly taken out from the slant surface using an inoculation loop, or a bacterial suspension was first prepared. Then, the bacterial cells were inoculated at the edge of the plate and excess bacterial cells were burned off. The plate was rotated at 60–70°, and gentle streaking was performed from left to right on the plate surface. The inoculation loop was sterilized by passing it through the flame outside the alcohol lamp after each turn. The area where the previous streak ended was lightly streaked without breaking the culture medium. After incubation, single colonies were observed and picked at the streaking site.

Slant passage preservation method (short-term preservation): By inhibiting the growth and reproduction of microorganisms using low temperatures, the shelf life of the culture is extended. The bacterial strains grown on slant culture medium were stored at 4–5°C in a refrigerator and transferred periodically, with a transfer performed every 3–6 months.

### 2.2 Screening of endophytic bacteria fermentation broth activity

#### 2.2.1 Preparation of fermentation broth

Revival of preserved endophytic bacterial strains (cultured on PDA solid medium at 28°C for 5 days), transferred to conical flasks containing Potato Dextrose Broth (PDB) liquid culture medium. Each conical flask contained 250 mL of medium, and the bacterial strains were cultured in a constant temperature shaking incubator at 28°C for 5–7 days. Stored at 4°C in a refrigerator for later use.

#### 2.2.2 Cytotoxicity experiment (MTT) sample preparation

The fermentation broth was placed into a blender, crushed, subjected to 10 min of ultrasonication, and then filtered using a filtration bottle.

#### 2.2.3 Metabolite sample processing

The filtrate was poured into a separating funnel and vigorously shaken for 10 min with ethyl acetate. This extraction was repeated three times, and the ethyl acetate layers were combined. The mixture was concentrated in a rotary evaporator (at 80°C under vacuum) to a small volume, transferred to an evaporation dish, and dried, yielding a crude extract.

The dried extract was dissolved in PBS and stored at 4°C under sterile conditions through a membrane filter (d = 0.22 μm, Minipore) for use in assessing anti-cancer potential and further research.

The metabolite profiling in this study adhered to the ConPhyMP framework, categorizing the extracts as Type C. High-resolution chemical fingerprints were generated to ensure reproducibility and compliance with phytochemical standards (ConPhyMP guidelines, 2023). Although Taoerqi has been listed in the Chinese Pharmacopoeia, Taoerqi endophytic fermentation metabolites belongs to Extract C in the ConPhyMP tool (https://ga-online.org/best-practice/). The chemical fingerprints of the metabolites and high-resolution mass spectrometry are in the Supplementary Materials.

#### 2.2.4 Cell sample processing

Recovery and proliferation of MDA-MB-231 cells (approximately 7 days for recovery-culture). Thaw the cryopreserved cell line rapidly by placing the cryovial in a preheated 37°C water bath, continuously shaking to ensure rapid melting of the liquid inside the vial. Approximately 1–2 min after complete dissolution of the liquid inside the cryovial, transfer the cell suspension into an EP tube containing 5 mL of DMEM high-glucose culture medium with 10% FBS. Centrifuge at 1000 rpm for 4 min, discard the supernatant, add an appropriate amount of fresh culture medium (containing 10% FBS, 1% penicillin-streptomycin in DMEM high-glucose culture medium, 14 mL), mix gently, and inoculate into a cell culture flask. Incubate at 37°C in a 5% CO_2_ cell culture incubator and change the culture medium approximately every 2 days.

The cells grow under constant conditions at 37°C. When the cell confluency reaches around 80%, passaging can be performed (usually takes about 3 days for proliferation to reach 80%).

#### 2.2.5 Cell passaging

When the cells in the culture flask reach approximately 80% confluency, discard the supernatant, wash gently three times with PBS, add 500 μL. of 0.25% trypsin, and incubate at 37°C for approximately 2–3 min until the cells become round and detach. Add an appropriate amount of complete culture medium to terminate cell digestion, gently pipette the cells, transfer the cell suspension into an EP tube containing 10% FBS in DMEM high-glucose culture medium, centrifuge at 1000 rpm for 5 min, discard the supernatant, add 5 mL of pre-prepared complete culture medium, mix gently, aspirate 50 μL. using a pipette for cell counting (using a hemocytometer):

① Wipe the cell counting chamber clean with a cotton ball dipped in 75% alcohol, let it air dry, and then cover it with a coverslip on one side.
② Pipette 50 μL of cell suspension from the gun head onto the edge of the coverslip of the cell counting chamber to fill the gap between the cell counting chamber and the coverslip.
③ Count the number of cells in the four large squares of the cell counting chamber under an inverted microscope (when encountering cells on grid lines, count only the cells to the right and below each grid line).
④ Calculate the cell density using the following formula: Cell density = (total number of cells in four large squares/4) × 10^4^ cells/mL

#### 2.2.6 MTT Experiment

Based on the cell concentration obtained in the previous step, adjust the cell concentration to 3 × 10^5^ cells/mL and seed them into a 96-well plate, adding 200 μL. per well, with 6 parallel wells per group. After the cells were cultured for 24 H, add 24 μL. of filtered metabolites solution (through a 0.22 μm filter) to each well. Set up a blank control group (only culture medium, no cells) and a control group (cells without metabolites). The last two rows were used as the color removal group (with culture medium and corresponding metabolites concentration). Incubate all groups at 37°C in a 5% CO_2_ cell incubator for 24, 48, and 72 H. Add MTT (5 mg/mL) at a volume of 20 μL per well (except for the slides and blank columns), incubate normally for 4 H, centrifuge, discard the supernatant, add 150 μL of DMSO per well (except for the slides and blank columns), shake for 10 min, and measure the absorbance at 490 nm wavelength on an enzyme immunoassay instrument - OD (A value). Calculate the inhibition rate using the formula: Inhibition rate (%) = (Control group A value - Experimental group A value)/Control group A value × 100%. Repeat the experiment 5 times.

### 2.3 Identification of dominant bacterial strains

#### 2.3.1 PCR amplification

Sequence amplification was performed using universal primers:

Bacteria: 27F/1492R primers
  27F: 5′-AGA​GTT​TGA​TCC​TGG​CTC​AG-3′
  1492R: 5′-GGT​TAC​CTT​GTT​ACG​ACT​T-3′
Fungi: ITS1/ITS4 primers
  ITS1: 5′-AGA​GTT​TGA​TCC​TGG​CTC​AG-3′
  ITS4: 5′-GGT​TAC​CTT​GTT​ACG​ACT​T-3′

##### 2.3.1.1 PCR reaction system

**Table:** 

10 × Ex Taq buffer	2.0 μL
5U Ex Taq	0.2 μL
2.5 mM dNTP Mix	1.6 μL
27 F/ITS1	1 μL
1492 R/ITS4	1 μL
DNA	0.5 μL
ddH_2_O	13.7 μL
Total volume	20 μL

##### 2.3.1.2 PCR reaction conditions

**Table:** 

95°C	5 min	
95°C	30 s	
56°C	30 s	25 circles
72°C	90 s	
72°C	10 min	

#### 2.3.2 Electrophoresis detection of amplified products

Electrophoresis was performed to preliminarily assess the amplification of PCR products for each sample.

#### 2.3.3 Sequencing of amplified products

Sequencing of the amplified products was carried out using the first-generation sequencing platform 3730. Each sample yielded.abl peak files and.seq sequence files. Generally, poor-quality sequences at both ends of the sequencing were removed by quality trimming. The quality-controlled paired-end sequencing results were assembled to obtain 16S rRNA or ITS sequences, saved in fasta format. Strain identification raw data are in Supplementary Material.

### 2.4 Influence of secondary metabolites on cancer cells

#### 2.4.1 Preparation of mRNA sequencing samples, WB samples, and PCR samples

Cells with a concentration of 3 × 10^5^ cells/mL were seeded into multiple 6-well plates with 2.5 mL per well. After 24 H of constant temperature incubation at 37°C under 5% CO_2_ conditions, 250 μL. of the corresponding metabolites solution was added to each well. After 8 H of incubation under 37°C constant temperature conditions with 5% CO_2_, samples were taken as follows:

Observe cell density and status (when cells grow to 80%–90% confluency), confirm cell status and integrity, and perform cell counting.

Remove the culture medium from the culture dish/bottle, wash gently three times with pre-cooled 1 × PBS, and centrifuge.

Discard the supernatant and resuspend the cell pellets with 1 mL TRIzol per 5 × 10^6^ cells, transfer into RNA-free cryogenic tubes, freeze rapidly in liquid nitrogen for 0.5 H, and then send the mRNA sequencing samples embedded in dry ice to Majorbio (Shanghai) for sequencing. WB and PCR test samples are stored at −80°C for less than 3 months.

#### 2.4.2 Sequencing experiment process

Eukaryotic mRNA sequencing is based on the Illumina Novaseq 6000 sequencing platform. It sequences all mRNA transcribed from specific tissues or cells of eukaryotes at a certain period. The sequencing experiment uses the Illumina TruseqTM RNA sample prep Kit method for library construction. The operation process diagram and instrument reagents are as follows:

ExtractTotaI RNA, Oligo dT Enriched RNA, Fragmented mRNA, Reverses synthetic cDNA, Connect adaptor, Illumina sequencing.

Extract total RNA from tissue samples and evaluate RNA concentration, purity, and integrity using Nanodrop 2000, agarose gel electrophoresis, and Agilent2100 for RIN value determination. Single library construction requires RNA total amount ≥1 ug, concentration ≥35 ng/μL, OD260/280 ≥ 1.8, and OD260/230 ≥ 1.0.

Enrich mRNA using Oligo dT that binds to the polyA tail at the 3′end of eukaryotic mRNA.

Fragment mRNA using fragmentation buffer to obtain fragments of around 300 bp.

Reverse transcribe cDNA using reverse transcriptase with random hexamers, forming stable double-stranded cDNA.

The process continues with steps such as adaptor ligation, library enrichment, PCR amplification, cluster generation, and sequencing on the Illumina platform (PE library, read length 2 × 150 bp).

#### 2.4.3 Bioinformatics analysis process

**FIGURE 1:**
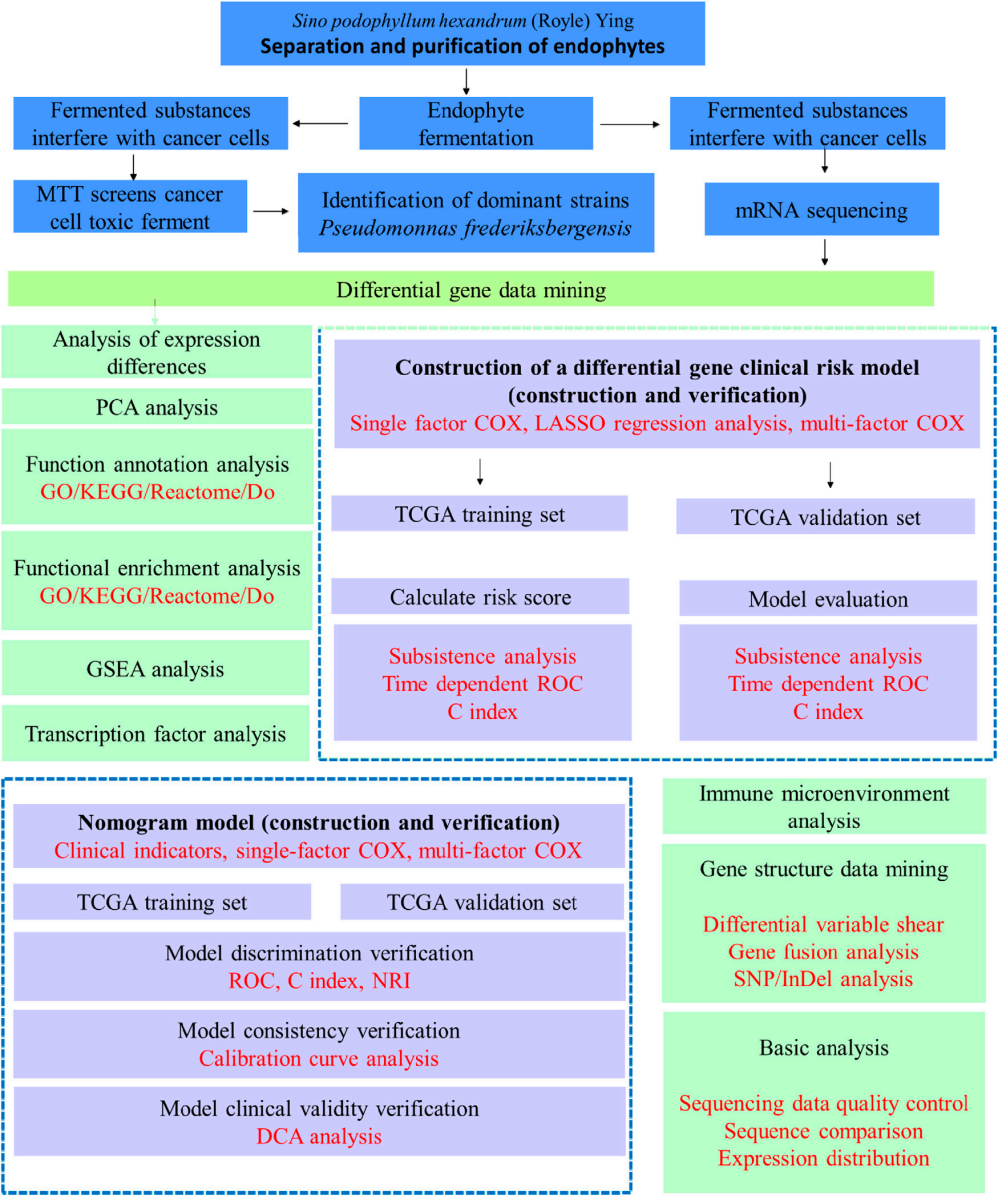
Bioinformatic analysis process.

The bioanalytical process is shown in Figure 1.

### 2.5 Targeted network construction

We predicted clinical prognosis outcomes by targeting specific miRNAs for genes (SARM1, RGS5, PROM2, BAG1). This involved screening for co-expressed mRNA and miRNA, leading to the identification of target miRNAs. The prediction of corresponding targeted lncRNAs was made, and a mRNA-lncRNA interaction network was drawn using Cytoscape.

### 2.6 Western blot (WB)

#### 2.6.1 Grouping

ZC group (blank control group): three parallel groups, each group was MDA-MB-231 cells without metabolites addition.

JL group (low dose group): three parallel groups, each group was MDA-MB-231 cells plus low dose *Pseudomonas frederiksbergensis* metabolite.

JM group (middle-dose group): three parallel groups, each with MDA-MB-231 cells plus middle-dose *P. frederiksbergensis* metabolite.

JH group (high dose group): three parallel groups, each with MDA-MB-231 cells plus high dose *P. frederiksbergensis* metabolite.

#### 2.6.2 Sample preparation

Add 150–250 μL of lysis buffer per 20 mg sample for complete lysis. Centrifuge the lysed sample at 4°C, 12000 g for 15 min, collect the supernatant, and store it in a −80°C freezer after protein quantification.

#### 2.6.3 Protein quantification

After constructing a standard curve, apply the sample, perform electrophoresis, transfer to a membrane, detect membrane proteins, block, incubate with antibodies, perform colorimetric detection, and analyze protein bands' grayscale values using ImageJ software.

### 2.7 Fluorescent quantitative PCR (qPCR)

#### 2.7.1 Total RNA extraction using the Trizol method

Remove cells from culture media, rinse twice with cold PBS, add 1 mL Trizol directly to a 3.5 cm culture dish for cell lysis. Add 0.2 mL chloroform (1/5 of the total volume of lysis buffer), close the cap, shake by hand for 15 s, and allow it to stand for 3 min. Centrifuge at 4°C, 12000 rpm for 15 min. The centrifuged sample separates into three layers, with RNA in the upper aqueous phase. Transfer 400 μL of the aqueous layer to a clean RNA-free centrifuge tube, add 0.8-fold isopropanol for RNA precipitation, mix thoroughly, incubate at −20°C for 15 min, and centrifuge at 4°C, 12000 rpm for 10 min. The white precipitate at the tube bottom is RNA. Wash the RNA precipitate with 1.5 mL 75% ethanol. Carefully remove the ethanol and air-dry the RNA. Dissolve the RNA precipitate in a dissolution solution and measure the RNA concentration.

#### 2.7.2 Nano drop RNA concentration measurement

The ratio of absorbance values at 260/280 nm and 260 nm and 280 nm assesses RNA purity, ideally around 2.0. The ratio of absorbance values at 260/230 nm indicates RNA purity secondary to 260/280 nm, generally between 1.8–2.2. The RNA values in this experiment were around 2.0 for both indicators.

#### 2.7.3 cDNA template synthesis and q-PCR experiment

Using a 20 μL system according to the reverse transcription kit’s instructions, select high-quality RNA to synthesize cDNA following the kit’s instructions. Perform q-PCR using the aforementioned cDNA as a template.

#### 2.7.4 Data processing

Analyze Real-time qPCR values using the 2-△△CT method. Use GraphPad PRISM 9.0 software for plotting. Statistical significance is confirmed when P < 0.05 (Tables 1, 2).

**TABLE 1:** Primer.

Gene	Primer	Primer Sequence (5′-3′)
β-actin	β-actin F	TGG​CAC​CCA​GCA​CAA​TGA​A
	β-actin R	CTA​AGT​CAT​AGT​CCG​CCT​AGA​AGC​A
SARM1	SARM1 F	AGG​CTG​TGC​TTA​CTT​TCA​ACG​GT
	SARM1 R	GTG​TCA​GAG​CCT​GCA​GAT​GAG​TC
RGS5	RGS5 F	TCT​CCT​CCA​GAA​GCC​AGA​CTC​AG
	RGS5 R	TGC​TTT​GCC​TTC​TCA​GCC​ATC​TT
PROM2	RGS5 F	TTT​GAG​TTT​GCA​GAC​ACC​CCA​GG
	RGS5 R	TCC​TTG​CAC​TGC​TGA​TAG​GCT​TG
BAG1	RGS5 F	CGA​CCT​TCA​TGT​TAC​CTC​CCA​GC
	RGS5 R	CCC​GGC​AAC​CAT​CTT​GTA​TTC​CA

Primers were synthesized by Shanghai Sangong Biological Engineering Co.

**TABLE 2:** Transcription conditions.

Clusters	Volumetric (μL.)	Amplification procedures	
cDNA	1	95°C–2 min	
Forward Primer (10 μM)	0.4	95°C–5 s	40 cycles
Reverse Primer (10 μM)	0.4	60°C–34 s	
2 × PerfectStart Green qPCR SuperMix	10	95°C–15 s	
Nuclease-free Water	8.2	60°C–1 min	Detected every 0.2°C rise
Total	20	95°C–15 s	

△△CT Method:

A = CT (Target Gene, Test Sample) - CT (Reference Gene, Test Sample).

B = CT (Target Gene, Control Sample) - CT (Reference Gene, Control Sample).

### 2.8 Determination of metabolites hemocompatibility

Preparation of metabolites solutions.

The extracted, concentrated, and filtered metabolites was diluted with normal saline to prepare 1%, 2%, and 5% solutions for use.

#### 2.8.1 Hemocompatibility assessment

Procedure: Whole blood was collected from New Zealand white rabbits and diluted with physiological saline. The diluted blood was stirred and centrifuged to prepare a 2% red blood cell (RBC) suspension. The RBC suspension was then mixed with the metabolites solutions of different concentrations in a 1:1 volume ratio and incubated at 37°C for 1 h. After incubation, the samples were centrifuged for 10 min, and the absorbance of the supernatant was measured at 540 nm using a microplate reader.

Controls:

Positive control (PC): 1% Triton X-100 solution.
Negative control (NC): physiological saline solution.

Hemolysis Rate Calculation:

$$\text{Hemolysis rate }\left(\%\right)=100\times \left(A-B\right)/\;\left(C-B\right)$$

Where:

• A: Absorbance of the test sample.
• B: Absorbance of the negative control.
• C: Absorbance of the positive control.

Groups: Positive control (PC), negative control (NC), and metabolites L, M, H.

#### 2.8.2 Coagulation function assessment

Procedure: Metabolites solutions at various concentrations were added to plasma at a 5% volume ratio, while the control group received plasma with 5% physiological saline instead. The mixtures were incubated in a thermostatic incubator at 37°C for 30 min. Coagulation parameters, including fibrinogen (Fg), activated partial thromboplastin time (APTT), prothrombin time (PT), and thrombin time (TT), were measured using an automated coagulation analyzer.

Groups: Positive control (PC), negative control (NC), and metabolites L, M, H. Each group consisted of 3 samples.

#### 2.8.3 Plasma protein determination

Procedure: Metabolites solutions at different concentrations were added to plasma at a 5% volume ratio. The control group received plasma with 5% physiological saline instead. The mixtures were incubated in a thermostatic incubator at 37°C for 30 min, followed by centrifugation at 3500 rpm for 10 min. The supernatants were collected for protein concentration measurement using commercial assay kits for total protein, albumin (Alb), immunoglobulin G (IgG), and fibrinogen.

Groups: Positive control (PC), negative control (NC), and metabolites L, M, H. Each group consisted of 3 samples.

#### 2.8.4 Dynamic coagulation time measurement

Procedure: Metabolites solutions at various concentrations were added to fresh blood at a 5% volume ratio. The control group received fresh blood with 5% physiological saline instead. After standing for specific time intervals (10, 20, 30, 40, and 50 min), the samples were placed in small beakers containing 15 mL of distilled water for 5 min. The absorbance of the solution was measured at 545 nm. For each time point, 3 samples were tested, and the average value was calculated.

Evaluation: Higher absorbance values indicate better anticoagulant performance.

Groups: Positive control (PC), negative control (NC), and metabolites L, M, H. Each group consisted of 3 samples, tested at 5 time points.

## 3 Results and discussion

### 3.1 MTT cell toxicity screening

Through MTT assay, dominant bacterial strains (number 16, 17) with certain anti-cancer activity were screened. The fermentation liquids of strains 16 and 17 were co-cultured with breast cancer cells for 24 H, 48 H, and 72 H. The inhibition rates are presented in Tables 3, 4. The inhibition rates of 2.5, 5, 10, and 20 μL of fermentation liquids from dominant strains (16, 17) co-cultured with breast cancer cells for 48 H are shown in Figure 1 below. At the same time, in order to test the intervention effect of the dominant strain 16 on different tumour cells, human breast cancer MDA-MB-231 cells, human breast cancer mda-mb-468 cells, human non-small cell lung cancer H1299 cells, human colon cancer SW480 cells, human non-small cell lung cancer A549 cells were selected as the intervention targets, and were diluted 50-fold from the original initial concentration and cultured with the corresponding tumour cells. The cells were diluted 50 times from the original initial concentration and cultured with the corresponding tumour cells. The cells were diluted 50 times from the original initial concentration and then co-cultured with the corresponding tumour cells for 24 h. The inhibition rates are shown in Supplementary Table 2.

**TABLE 3:** Cell inhibition rate of sample 16/17.

Group	A490		Inhibition rate	
	24 H	72 H	24 H	72 H
Medium + cells	0.4954 ± 0.0336	1.3849 ± 0.0281		
Medium	0.0591 ± 0.0048	0.0900 ± 0.0038		
Medium + 16	0.075 ± 0.0031	0.1208 ± 0.0023		
Medium + 17	0.0859 ± 0.0058	0.1440 ± 0.0027		
Medium + 16 + cells	0.3236 ± 0.0355^**^	0.6642 ± 0.0297^**^	43.11%	58.04%
Medium + 17 + cells	0.3274 ± 0.0919^*^	0.8562 ± 0.0352^**^	44.63%	44.99%

Compared with Medium + cells *p < 0.01, **p ≪ 0.001.

**TABLE 4:** Inhibition of different kinds of tumor cells by fermented extracts of *Pseudomonas frederiksbergensis*.

Cell types	A490				Inhibition rate (24H)
	Medium + cells	Medium	Medium + metabolites	Medium + metabolites + cells	
MDA-MB-231	1.0684 ± 0.0346	0.0577 ± 0.0025	0.1215 ± 0.0094	0.7678 ± 0.0065	36.07%
MDA-MB-468	1.0117 ± 0.0617	0.0525 ± 0.0011	0.0879 ± 0.0008	0.8006 ± 0.0787	25.70%
H1299	1.3556 ± 0.0534	0.0591 ± 0.0064	0.0956 ± 0.0191	0.7803 ± 0.0763	47.19%
SW480	1.1006 ± 0.0976	0.0546 ± 0.0012	0.1067 ± 0.0034	0.9305 ± 0.1254	21.24%
A549	1.1545 ± 0.0876	0.0517 ± 0.0037	0.1038 ± 0.0003	0.7783 ± 0.0236	39.11%

Re-fermentation of dominant bacterial strains, when co-cultured with cancer cells for 24 H, 48 H, 72 H, still exhibited satisfactory inhibition rates as presented in Table 3.

**FIGURE 2:**
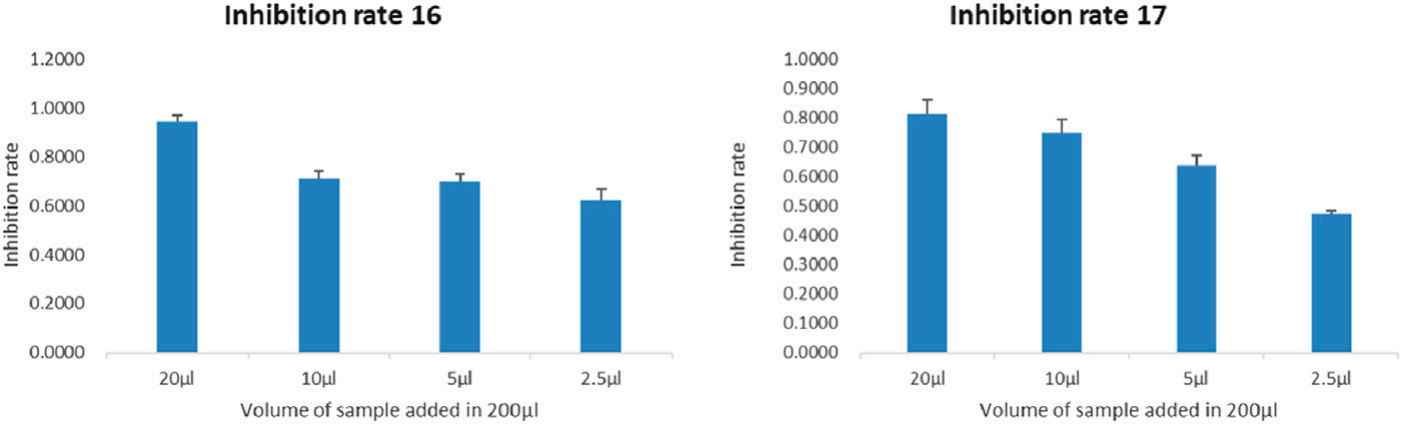
Concentration-dependent experiment of samples 16 and 17 for 48 H (as the dosage increases, the inhibition rate gradually increases) (the horizontal axis represents the dosage, and the vertical axis represents the cell inhibition rate).

The dominant strains' secondary metabolites (16 & 17) exhibited potent anticancer cytotoxicity. Notably, this cytotoxic effect intensified with increasing concentration and prolonged exposure to the cancer cells (Figure 2).

As shown in Table 4, when the dominant strain 16 was selected and diluted 50 times to intervene with different tumour cells, the tumour cell inhibition remained significant.

### 3.2 Morphology and identification results of bacterial strains

Sequencing was performed using the 3730 first-generation sequencing platform on amplified products, yielding peak graph files in.abl format and sequence files in.seq format for each sample. Generally, the sequence quality at both ends of sequencing is lower, so trimming was performed to remove low-quality sequences from both ends. The double-ended sequencing results after quality control were assembled to obtain 16S rRNA or ITS sequences, which were saved in fasta format.

Species Confirmation by Database Alignment: Each sample’s assembled sequence was aligned with databases, and species were determined based on coverage, similarity, and alignment results. The species with the highest alignment score was preliminarily identified as *P. frederiksbergensis*.

Phylogenetic Analysis: Approximately 15–20 different microbial species closely related to the samples were selected by database alignment. Using MEGA, a phylogenetic tree depicting the evolutionary relationships within a larger population was constructed. This analysis helped determine the sample’s evolutionary relationship and classification status within a broader population.

**FIGURE 3:**
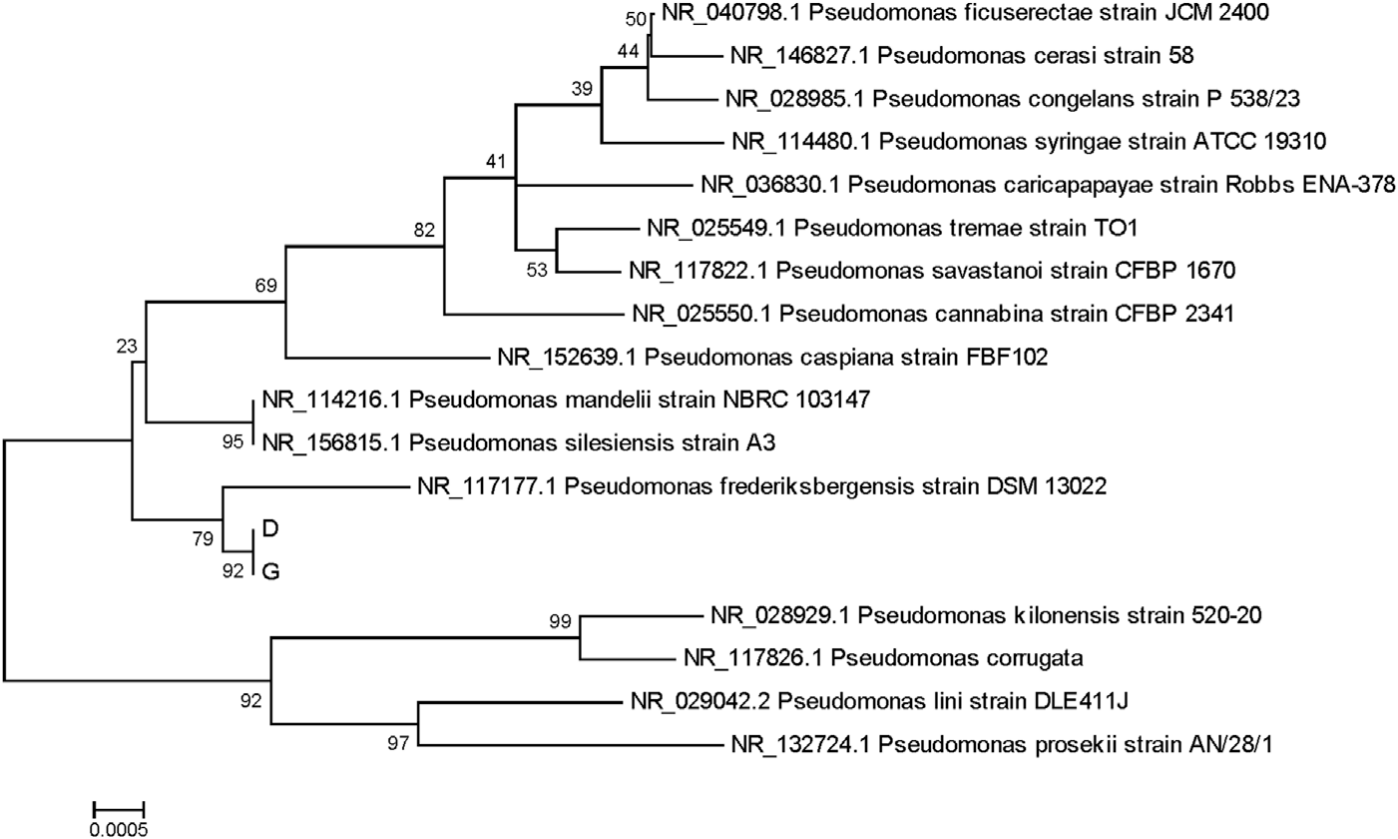
Phylogenetic tree of samples D (16), G (17).

**FIGURE 4:**
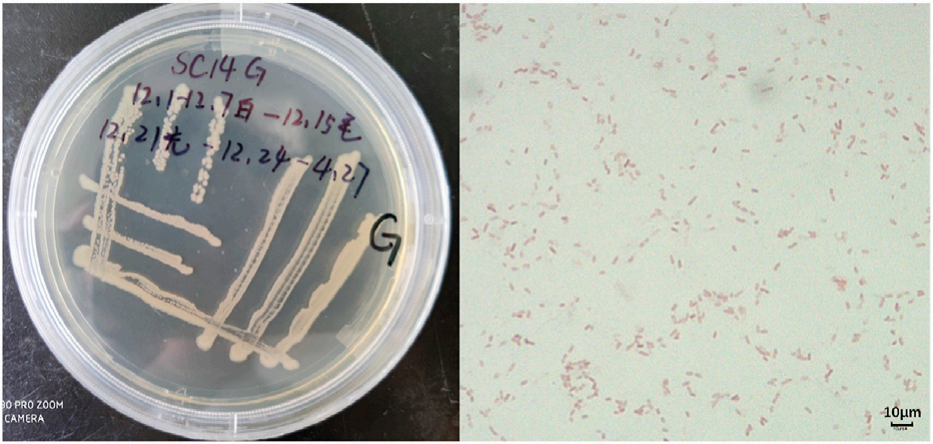
Appearance as well as microstructure of endophytic colonies.

Ultimately, both strains were confirmed as *P. frederiksbergensis* (Andersen et al., 2000; Kumar et al., 2019; Ruiz et al., 2021) (Figures 3, 4).

### 3.3 Differential gene data mining

Sequencing Data Statistics and Grouping: Transcriptome sequencing was conducted for 6 samples in total, with 3 samples in each of the two groups (JWTQ17 treatment group and KB blank group), resulting in a total of 46.48 Gb Clean Data. Each sample’s Clean Data reached 6.88 Gb or more, with Q30 base percentage above 94.02%.

Reference Genome Alignment: Using *Homo sapiens* as the reference gene source (GRCh38 version, reference genome source: http://asia.ensembl.org/Homo_sapiens/Info/Index), the Clean Reads of each sample were separately aligned to the designated reference genome. Alignment rates ranged from 96.51% to 97.02%.

Expression Level Analysis: A total of 25,301 expressed genes were detected in this analysis, comprising 25,028 known genes and 273 new genes. Additionally, 114,041 expressed transcripts were identified, consisting of 105,442 known transcripts and 8,599 new transcripts.

Differential Expression Analysis: Through quantitative analysis, we identified genes with varying expression levels between groups. This differential expression is critical for understanding the functional impact of these genes in different sample groups. Based on quantitative expression results, inter-group differential gene analysis was conducted using DESeq2 software with a screening threshold of |log2FC|≥1 and padjust <0.05, resulting in: Total genes: 2,153, Upregulated: 1,254, Downregulated: 899.

Inter-sample Correlation Analysis: We analyzed the correlation among biological replicates to validate the consistency with the experimental design. This step ensures the reliability of our differential gene analysis, providing a foundational reference. A higher correlation coefficient closer to 1 signifies higher similarity in gene/transcript expression levels between samples, indicating better inter-sample correlation.

**FIGURE 5:**
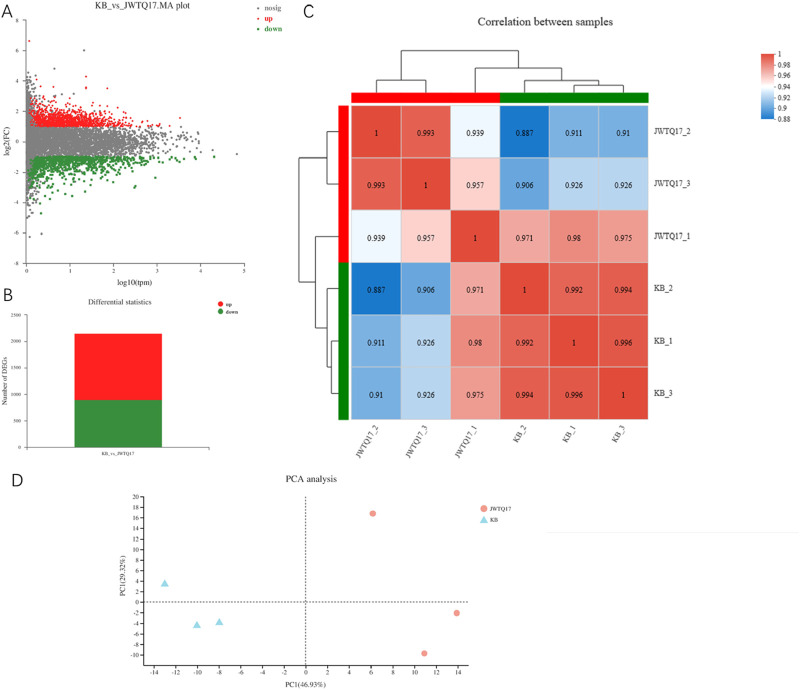
**(A)** displays a volcano plot illustrating the differential expression of genes based on selection criteria ∣log2FC∣≥1 and P < 0.05. The horizontal axis represents the expression values obtained from contrasting groups, while the vertical axis shows the changes in gene expression between the differential comparison groups derived from differential expression analysis. The values on both axes have undergone logarithmic transformation. Each point in the plot represents a specific gene, with red dots indicating significantly upregulated genes, green dots indicating significantly downregulated genes, and gray dots representing non-significant differentially expressed genes. Upon mapping all genes, it becomes apparent that genes positioned higher in the plot are upregulated, whereas those situated lower are downregulated, indicating a more significant expression difference. **(B)** presents a statistical graph depicting the differential expression levels (total DEG: 2153 differentially expressed genes; (3) up: 1254 upregulated differentially expressed genes; (4) down: 899 downregulated differentially expressed genes). **(C)** shows a heatmap illustrating the correlation between samples, where JWTQ17-1/2/3 represents cancer cells treated with NO.17, and KB-1/2/3 denotes untreated cancer cells. The left and upper sides display the clustering of various samples/groups, with different colored squares indicating the degree of correlation between two samples/groups. Larger values indicate higher correlation between the two, signifying better biological replicability between the samples. **(D)** displays a principal component analysis (PCA) plot of samples after dimensionality reduction. The distance between sample points represents their similarity, with closer distances indicating higher similarity between samples. The horizontal axis represents the contribution of the first principal component (PC1) in distinguishing samples, while the vertical axis represents the contribution of the second principal component (PC2) in distinguishing samples.

PCA Analysis between Samples: Principal Component Analysis (PCA) reduces data complexity and delves into the relationships and variations among samples. By reorganizing variables into new, unrelated comprehensive variables (i.e., principal components), PCA ranks factors by importance, typically discarding less impactful factors to simplify data. PCA analysis effectively separates the metabolites-treated sample (JWTQ17) from the untreated sample (KB), demonstrating significant impact of the metabolites (JWTQ17) on cancer cell growth (Figure 5).

### 3.4 Functional annotation analysis of differential genes

**FIGURE 6:**
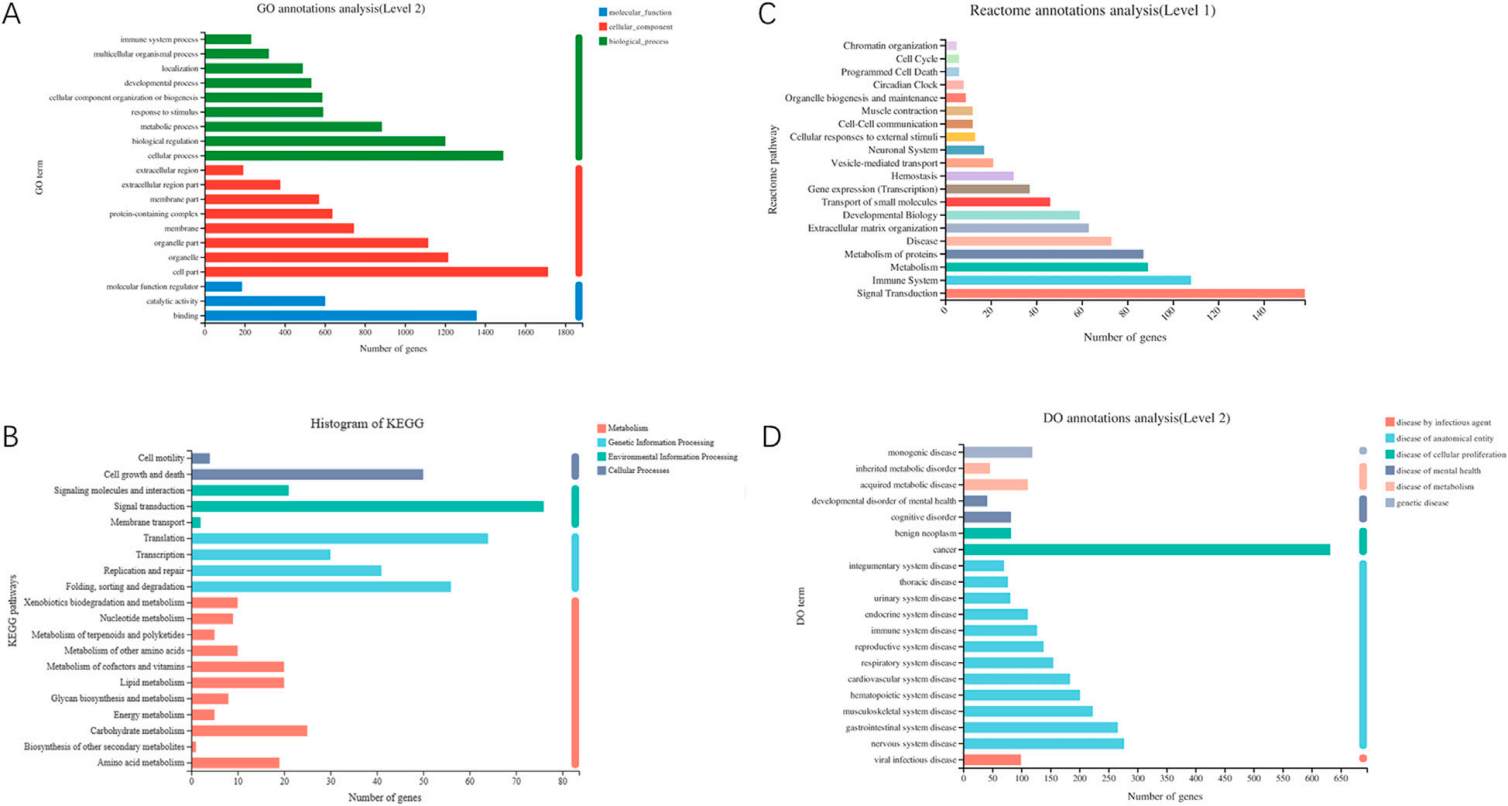
**(A)** GO classification statistical chart (Each gene has multiple GO functions, thus, the total percentage sums to greater than 1). **(B)** KEGG classification statistical chart (The vertical axis represents the names of KEGG metabolic pathways; the horizontal axis shows the quantity of genes annotated to each pathway. KEGG metabolic pathways are classified into seven major categories: Metabolism, Genetic Information Processing, Environmental Information Processing, Cellular Processes). **(C)** Reactome classification statistical chart (The vertical axis represents the names of Reactome metabolic pathways; the horizontal axis indicates the number of genes annotated to each pathway). **(D)** DO classification statistical chart (The vertical axis shows the second-level terminology of DO; the horizontal axis displays the quantity of genes matching this second-level classification. The colors denote categories such as disease by infectious agent, disease by anatomical entity, disease of cellular proliferation, disease of mental health, disease of metabolism, genetic disease). Note: DO and Reactome only support gene-level analysis, and DO analysis is only applicable to projects focusing on human research species.

**FIGURE 7:**
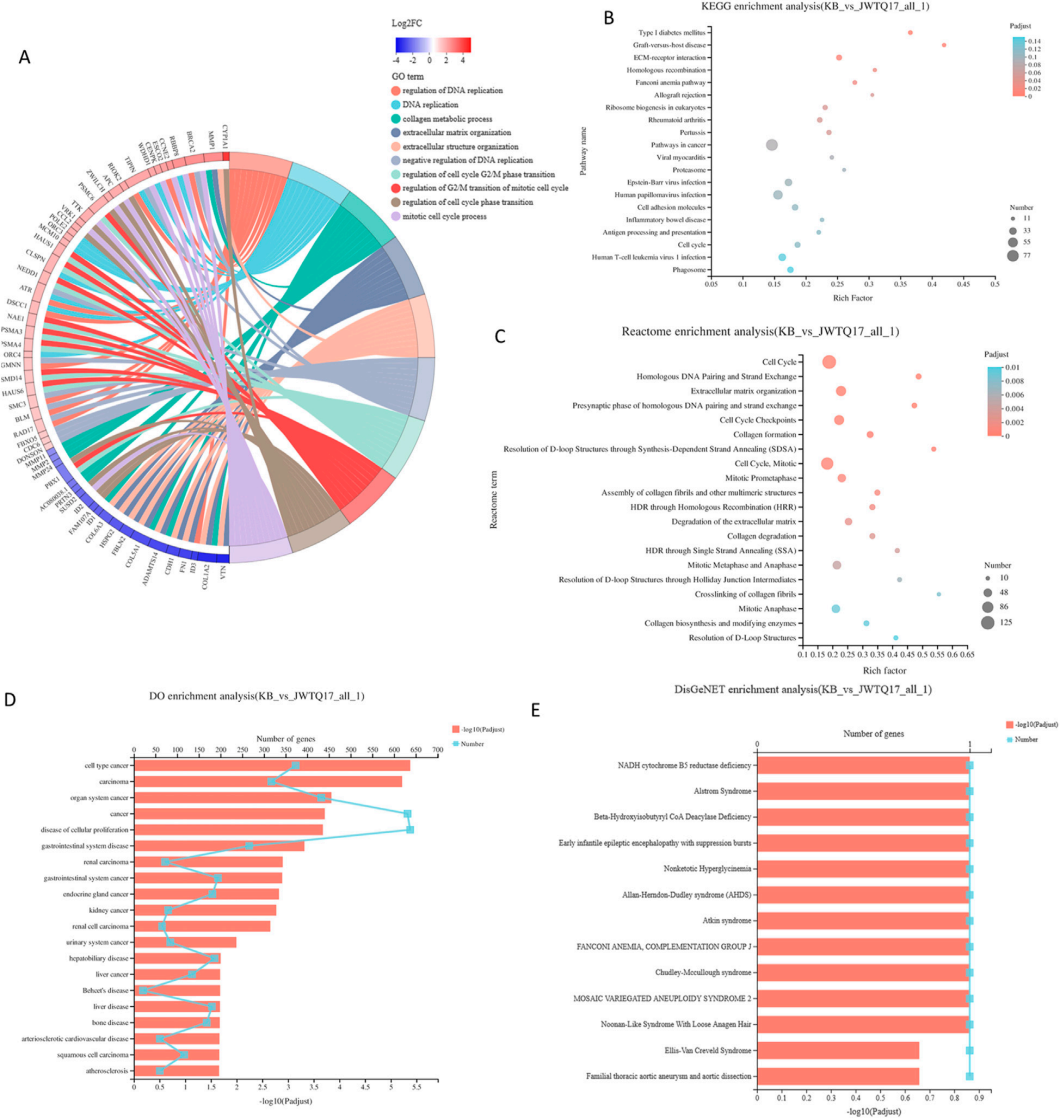
**(A)** GO enrichment chord diagram. The right side displays significant enrichment information for Terms/Pathways of differential genes, while the left side represents genes included in that Term/Pathway, arranged in descending order of log2FC. A higher log2FC indicates a greater expression difference for upregulated genes, whereas a smaller logFC signifies a larger expression difference for downregulated genes. When log2FC approaches 0, it indicates a smaller differential expression multiple for genes. **(B)** KEGG enrichment analysis results - Bubble plot (The vertical axis represents KEGG pathway names, while the horizontal axis represents the ratio of genes enriched in the KEGG pathway (Sample number) to the number of annotated genes (Background number). A higher Rich factor indicates a greater level of enrichment. The size of the dots indicates the quantity of genes in the respective KEGG pathway, and the colors correspond to different P-adjusted ranges. The top 20 enriched results are shown under the premise of P-adjust <0.5). **(C)** Reactome enrichment analysis results - Bubble plot (The vertical axis displays Reactome pathway names, while the horizontal axis shows the ratio of genes enriched in the Reactome pathway (Sample number) to the number of annotated genes (Background number). The size of the dots denotes the number of genes in each Reactome pathway, and the colors represent different P-adjusted ranges. The top 20 enriched results are displayed under the premise of P-adjust <0.5). **(D)** DO enrichment analysis results - Bar plot (with lines) (The vertical axis represents DO terms; the bottom horizontal axis indicates the quantity of genes matching each DO term, corresponding to different points on the line. The top horizontal axis represents the significance level of enrichment, corresponding to the height of the bars. The smaller the P-adjust, the larger the -log10(P-adjust) value, indicating more significant enrichment of the DO term. The top 20 enriched results are shown under the premise of P-adjust <0.5). **(E)** DisGeNET enrichment analysis results - Bar plot (with lines) (The vertical axis represents DisGeNET terms; the bottom horizontal axis indicates the quantity of genes matching each DisGeNET term, corresponding to different points on the line. The top horizontal axis represents the significance level of enrichment, corresponding to the height of the bars. The smaller the P-adjust, the larger the -log10(P-adjust) value, indicating more significant enrichment of the DisGeNET term. The top 20 enriched results are shown under the premise of P-adjust <0.5). Note: DO and Reactome support gene-level analysis only, and DO and DisGeNET analysis can only be conducted for projects focusing on human research species.

This section involves functional analysis of genes within the differential gene set, including GO, KEGG, Reactome, and DO classification annotations (Figures 6, 7).

### 3.5 Functional enrichment analysis of differential genes

This section involves conducting functional enrichment analysis of genes within the differential gene set, determining the primary functionalities or major involvement in metabolic pathways of this gene set. This includes GO enrichment analysis, KEGG enrichment analysis, Reactome enrichment analysis, DO enrichment analysis, and DisGeNET enrichment analysis.

Based on the sequencing results, the Cell cycle pathway was significantly enriched in multiple enrichment methods. It is suggested that the study of *P. frederiksbergensis* metabolites should focus on the target of Cell cycle action in the future scientific research. (Piezzo et al., 2020; Butt et al., 2008; Yin et al., 2023).

### 3.6 Construction of differential gene clinical risk model

Data Source: Transcriptome Count data and clinical information of breast cancer were downloaded from TCGA database through Sangerbox 3.0 (http://vip.sangerbox.com). This dataset included 113 normal or adjacent tissue samples and 1091 breast cancer patient samples, providing a total of 60489 gene targets. Intersection of TCGA data targets with differentially expressed genes (2153) resulted in 2133 common genes.

COX Regression Model Construction: The COX regression model utilized survival outcomes and survival time as dependent variables, analyzing multifactorial effects on survival duration. Using the common gene set for Cox risk score model data, we conducted Univariate Cox regression analysis, LASSO regression analysis (Least Absolute Shrinkage and Selection Operator), and multifactorial Multivariate Cox regression analysis to construct the COX risk score model.

To observe the relationship between the common gene set expression and prognosis, we randomly divided 1089 breast cancer samples into two groups: training set (700 samples) and validation set (389 samples). Regarding the expression levels of common genes and clinical survival data, we performed single-factor COX regression analysis on the training set, resulting in 114 genes from uniCox analysis, 45 genes from LASSO analysis, and 27 genes from multiCox analysis.

**FIGURE 8:**
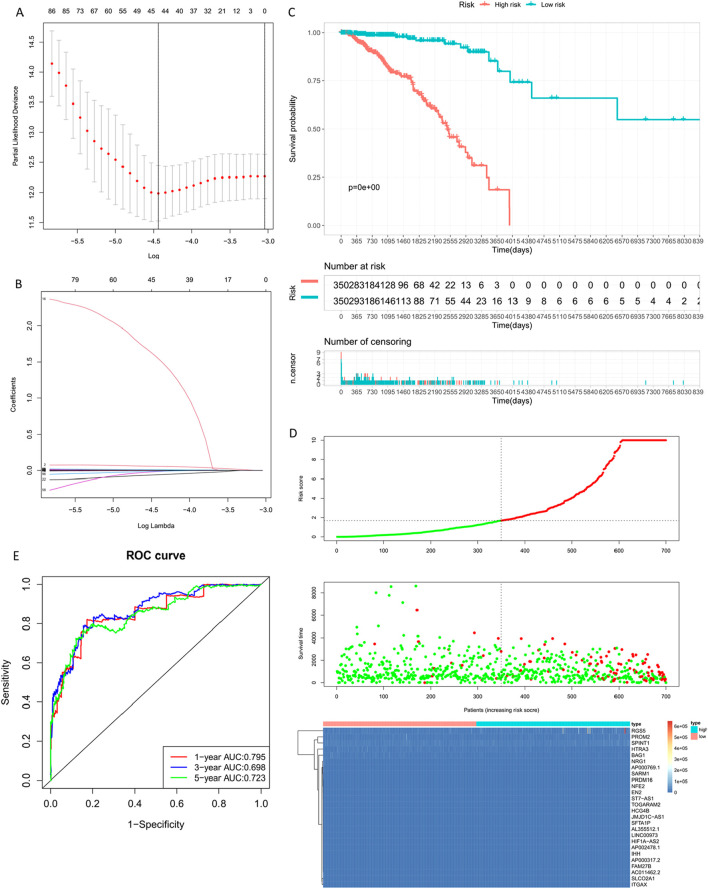
Building the Training Set Risk Assessment Model. **(A)** Standard deviation distribution of models at different lambdas. **(B)** Gene coefficient trajectory at different lambdas. **(C)** Prognostic KM curve for the high and low-risk groups; the high-risk group shows significantly shorter survival than the low-risk group. **(D)** RiskPlot curve, where the number of deceased patients is notably higher in the high-risk group. **(E)** Model evaluation - Time-dependent ROC curve (1-year, 3-year, 5-year).

Following LASSO regression analysis using ten-fold cross-validation and summarizing the dimensional reduction results, counting the frequency of each probe’s appearance, the combinations with the highest frequencies were observed. Finally, these genes (Table 5) showed trajectories concerning the coefficient changes with different lambdas in Figure 8, while the standard deviation distribution of different lambdas is depicted in Figure 7. Further analysis via KM curve demonstrated that this gene set can significantly differentiate between high and low-risk groups.

**TABLE 5:** Multivariate Cox gene based signature.

Id	coef	HR	HR.95L	HR.95H	*p* value
AP000317.2	0.071261	1.073861	1.029749	1.119863	0.000869
LINC00973	0.011253	1.011317	1.006751	1.015902	1.09E-06
TOGARAM2	−0.00818	0.991849	0.979621	1.004231	0.196012
HIF1A-AS2	0.021367	1.021597	1.012218	1.031061	5.60E-06
PRDM16	0.000887	1.000887	0.999918	1.001858	0.072932
EN2	0.001851	1.001853	0.999461	1.004249	0.128993
FAM27B	2.205989	9.079231	2.891796	28.50562	0.000157
PROM2	5.20E-05	1.000052	1.00001	1.000094	0.014641
AC011462.2	−0.17244	0.841606	0.731145	0.968755	0.016299
ST7-AS1	−0.00356	0.996444	0.992411	1.000493	0.085092
SLCO2A1	0.000169	1.000169	1.000043	1.000296	0.008789
AL355512.1	0.007062	1.007087	0.998261	1.015992	0.115851
SPINT1	5.22E-05	1.000052	1.000022	1.000083	0.00075
HCG4B	−0.01031	0.98974	0.976006	1.003667	0.148036
HTRA3	−4.18E-05	0.999958	0.999906	1.000011	0.119843
NFE2	−0.00424	0.99577	0.992823	0.998726	0.005067
BAG1	−0.00016	0.999836	0.999749	0.999923	0.000232
AP002478.1	−0.41836	0.658128	0.542068	0.799038	2.37E-05
NRG1	−0.00128	0.998725	0.997958	0.999493	0.001139
JMJD1C-AS1	0.00839	1.008426	1.004422	1.012446	3.57E-05
SFTA1P	0.01152	1.011587	1.005774	1.017433	8.92E-05
ITGAX	−0.0002	0.999803	0.999553	1.000053	0.123341
IHH	−0.08086	0.922325	0.846898	1.00447	0.06324
SARM1	−0.00086	0.999141	0.998479	0.999803	0.011033
RGS5	5.63E-06	1.000006	1.000002	1.00001	0.004817
AP000769.1	0.000308	1.000308	1.000082	1.000534	0.007559

By calculating the risk scores of each sample based on the expression levels of the training set samples, we plotted the distribution of RiskScore for samples as shown in Figure 8. Calculated risk scores to evaluate the association between gene expression and potential molecular mechanisms in breast cancer. From the figure, it is evident that samples with higher risk scores had noticeably shorter Overall Survival (OS) compared to those with lower scores, indicating that samples with higher RiskScore had a worse prognosis. Furthermore, using the R package ‘timeROC' for RiskScore, we conducted ROC analysis for prognosis classification, demonstrating high AUC values of around 0.7 for one, three, and 5-year prognostic predictions (Jia et al., 2023; Kang et al., 2023).

**FIGURE 9:**
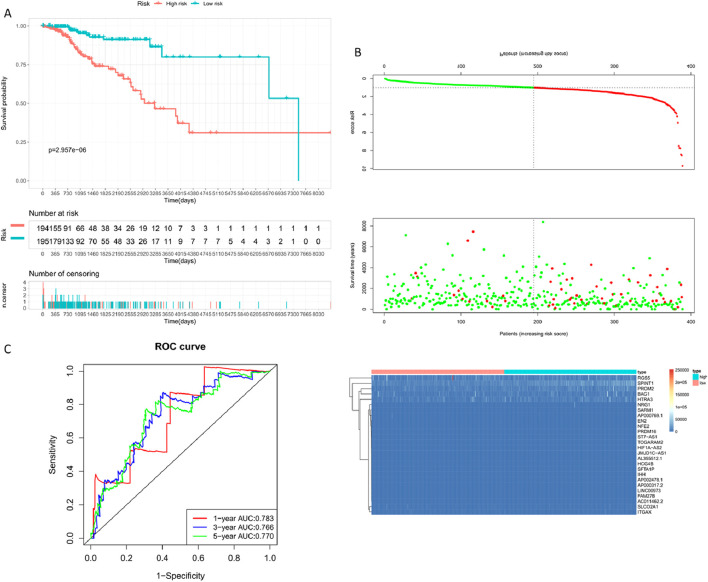
Constructing the Validation Set Risk Assessment Model. **(A)** Prognostic KM curve for the high and low-risk groups; the high-risk group shows significantly shorter survival than the low-risk group. **(B)** RiskPlot curve; the high-risk group exhibits a noticeably higher number of deceased patients than the low-risk group. **(C)** Model evaluation - Time-dependent ROC curve (1-year, 3-year, 5-year).

Breast cancer targets for which *P. frederiksbergensis* metabolites have a clinically significant prognostic effect were screened by risk scoring modeling, and once again, the potential therapeutic effect of the metabolites on these breast cancer targets was verified by validation set risk assessment modeling (Zhu et al., 2023) (Figure 9, Table 6).

**TABLE 6:** Riskscore C index of Training set and validation set.

Group	c.Index	se	Lower	Upper	*p*.value
Training set	0.8350977	0.02567499	0.7847756	0.8854197	6.229114e-39
Validation set	0.7123169	0.03755439	0.6387116	0.7859221	1.571368e-08

Risk scores were calculated for each patient and patients were categorized into high-risk and low-risk groups using the median cutoff value.

Risk score = AP000317.2 × 0.071261 + LINC00973 × 0.011253 - TOGARAM2 × 0.00818 + HIF1A-AS2 × 0.021367 + PRDM16 × 0.000887 + EN2 × 0.001851 + FAM27B × 2.205989 + PROM2 × 5.20E-05 - AC011462.2 × 0.17244 - ST7AS1 × 0.00356 + SLCO2A1 × 0.000169 + AL355512.1 × 0.007062 + SPINT1 × 5.22E-05 - HCG4B × 0.01031 - HTRA3 × 4.18E-05 - NFE2 × 0.00424 - BAG1 × 0.00128 - AP002478.1 × 0.41836 - NRG1 × 0.00128 + JMJD1CAS1 × 0.00839 + SFTA1P × 0.01152 - ITGAX × 0.0002 - IHH × 0.08086 - SARM1 × 0.00086 + RGS5 × 5.63E-06 + AP000769.1 × 0.000308.

### 3.7 Construction of nomogram model

Based on the risk model established earlier, we identified prognostic biomarkers. In addition to assessing the impact of metabolitess on biomarkers through ROC curves and survival analysis, combining biomarkers with clinical features enables a comprehensive prediction of patient survival, thus leaning towards personalized medicine in the metabolites treatment process (Mulé, 2023; Xia et al., 2023).

**FIGURE 10:**
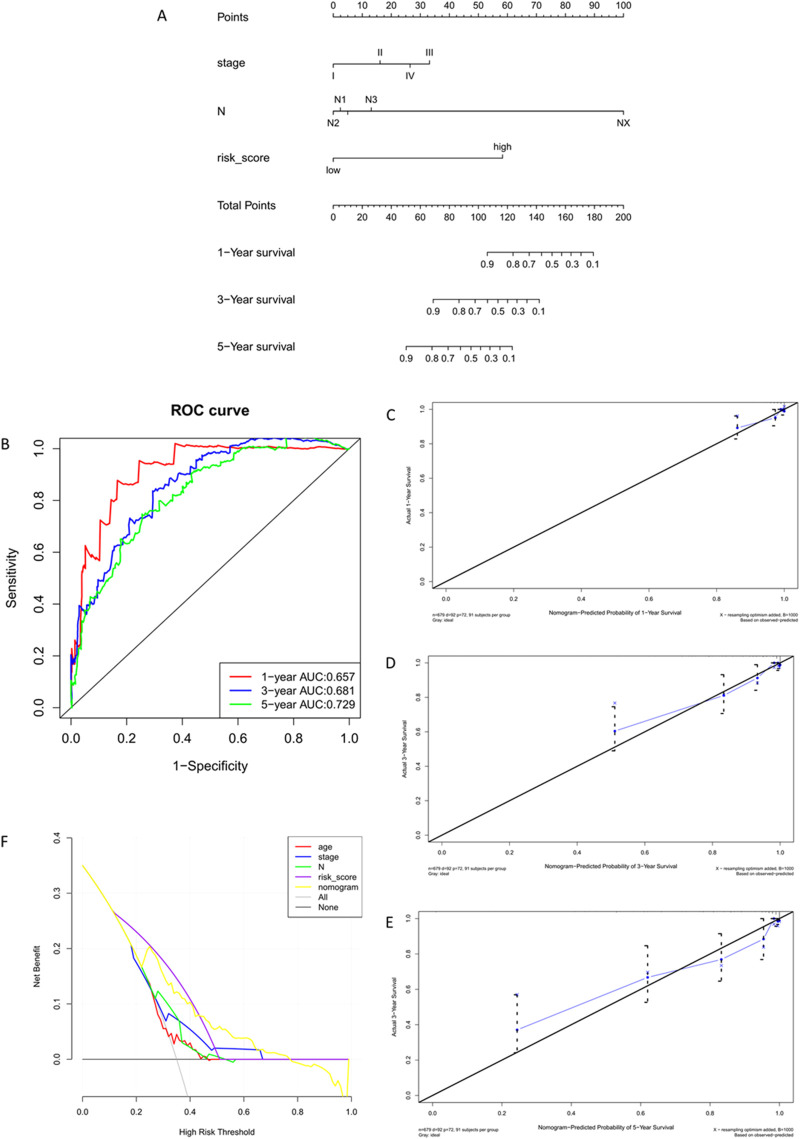
Training Set Nomogram Model Construction and Validation. **(A)** Nomogram column chart. **(B)** Model evaluation - Model discriminative ability validated by ROC curve. **(C–E)** Clinical consistency validation - 1, 3, 5-year calibration plots. **(F)** Clinical limited evaluation - Decision Curve Analysis, DCA.

The Nomogram integrates multiple predictive indicators based on the results of the multifactorial COX regression analysis. It visually displays the relationship between clinical data and survival in graphical form (Figure 10A). After constructing the Nomogram model, ROC curves are used to evaluate the model’s discriminative ability (Figure 10B). Calibration plots assess the model’s consistency. The closer the curve fits the diagonal line, the better the predictive value of the model (Figures 10C–E) illustrates the calibration plots for 1/3/5 years, showing good fitting.

ROC curves only evaluate the specificity and sensitivity of the model, and false negatives may still exist in clinical settings. Decision Curve Analysis (DCA) evaluates the effectiveness of clinical indicators. In DCA curves, ‘All' and ‘None' represent two extreme situations. The straight line represents no intervention for everyone, with a net benefit of 0. The diagonal line represents all samples being positive, indicating a negative slope for everyone receiving intervention (Figure 10F).

The prognostic effect of *P. frederiksbergensis* metabolites on the treatment of breast cancer patients was further investigated by survival prognostic modeling, and 5-, 3-, and 1-year survival rates were all improved.

**FIGURE 11:**
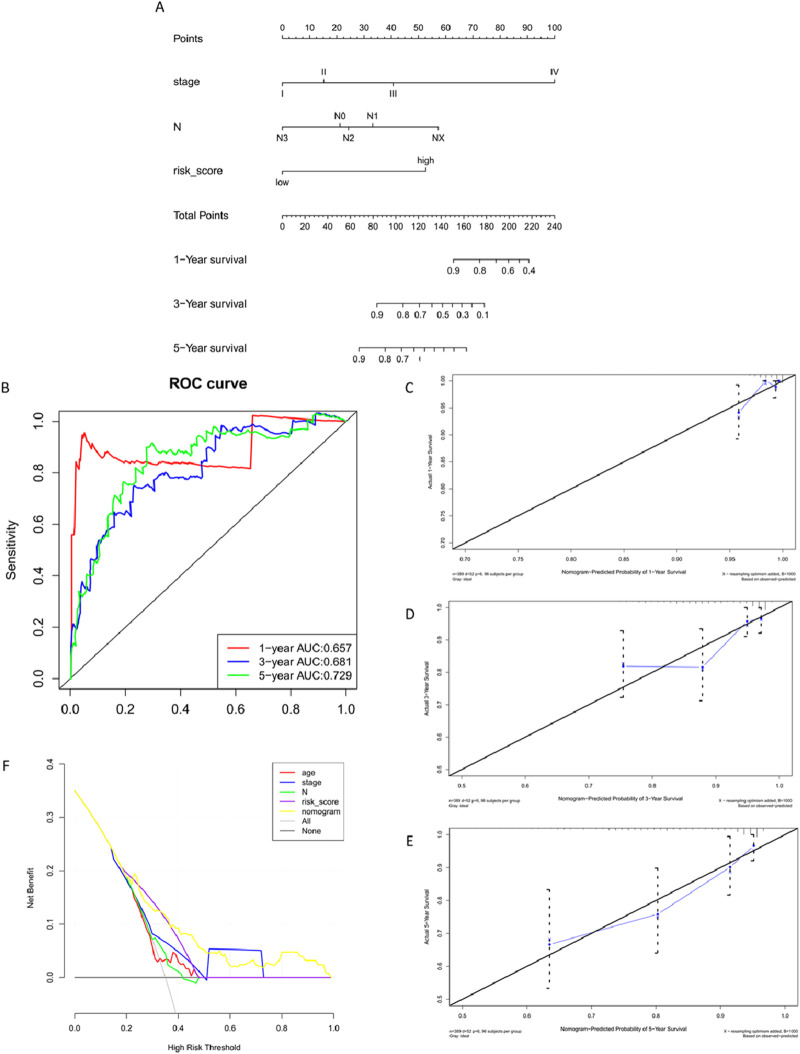
Validation Set Nomogram Model Construction and Validation. **(A)** Nomogram column chart. **(B)** Model evaluation - Model discriminative ability validated by ROC curve. **(C–E)** Clinical consistency validation - 1, 3, 5-year calibration plots. **(F)**. Model evaluation - Clinical limited evaluation - Decision Curve Analysis, DCA.

Validating the validation set data using the Nomogram model yielded results similar to those of the training set, confirming that the Nomogram model reflects clinical validation of risk scores (Figure 11) (Table 7).

**TABLE 7:** Nomogram C index of Training set and validation set.

Groups	c.Index	se	Lower	Upper	*p*.Value
Training set	0.8358683	0.0210686	0.7945746	0.877162	3.25621e-57
Validation set	0.7888763	0.04339162	0.7038303	0.8739223	2.786748e-11

### 3.8 Analysis of gene expression characteristics of CIBERSORT immune cell subtypes

Based on the principle of linear support vector regression, convolution of the expression matrix of human immune cell subtypes is performed. This algorithm is based on a known reference set and provides gene expression feature sets for 22 immune cell subtypes (Li et al., 2023; Zhang et al., 2023; Luo et al., 2019).

**FIGURE 12:**
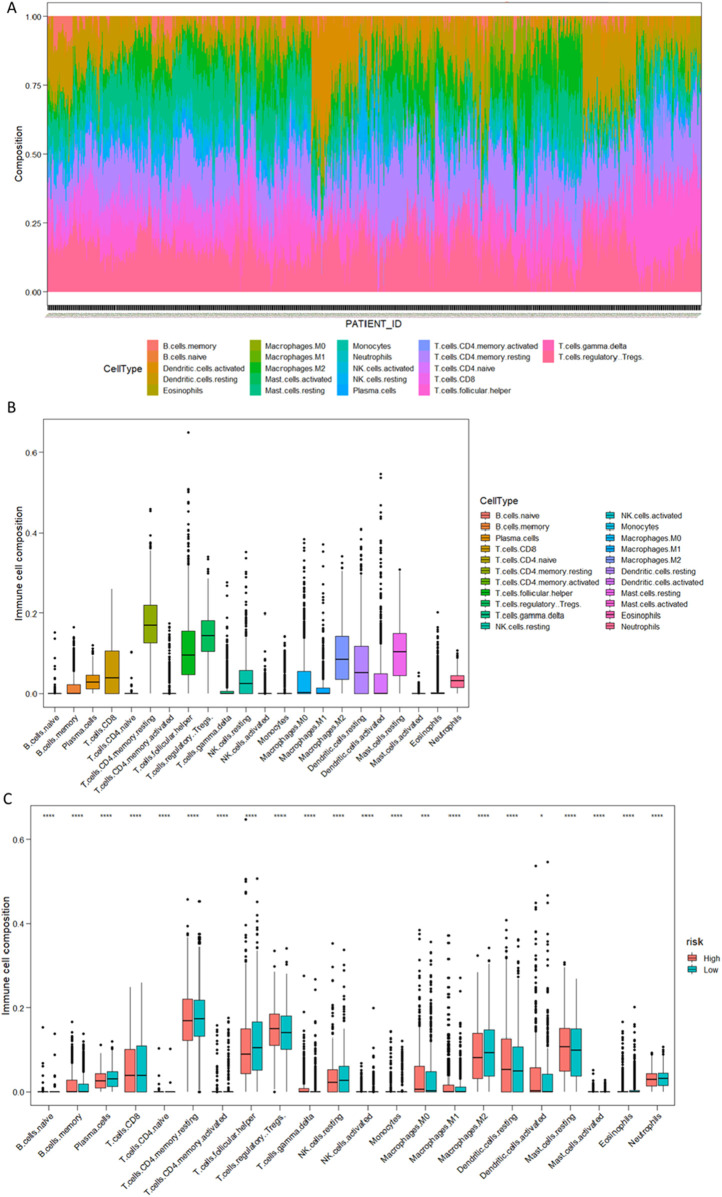
**(A)** Overview of 22 immune cell types (barplot). **(B)** Overview of 22 immune cell types (boxplot). **(C)** Comparison of the distribution of 22 immune cell types in High/Low Risk groups (****P < 0.00001, ***P < 0.0001, *P < 0.01).

We used expression profile data from 1089 breast cancer patients, applying linear support vector regression to deconvolute the expression matrix of human immune cell subtypes. Analyzing the gene expression feature sets of 22 immune cell subtypes based on the known reference set, this method is superior in deconvolution analysis for unknown substances and matrices containing similar cell types. There were significant differences observed in the B cells memory, T cells CDB, T cells CD4 memory resting, NK cell activated, Macrophages M1, Dendritic cells resting, Dendritic cells activated, Mast cells resting, Eosinophils, and Neutrophils between the High and Low Risk groups. This indicates significant differences in the tumor cell immune microenvironment between the High Risk/Low Risk groups, further suggesting that metabolites-influenced gene differences also have an impact on the tumor microenvironment in the High Risk/Low Risk groups (Figure 12).

### 3.9 Gene set enrichment analysis (GSEA)

Gene set enrichment analysis aims to assess the distribution trend of the gene set of differentially expressed genes in a sorted gene expression profile related to the phenotype, to determine its contribution to the phenotype.

Gene Set Enrichment Analysis (GSEA) involves using pre-defined gene sets, usually derived from functional annotations or previous experimental results (contained in MSigDB). Genes are ranked based on their differential expression in two sample classes, and the pre-defined gene set is then tested to determine whether it clusters at the top or bottom of the ranked list.

**FIGURE 13:**
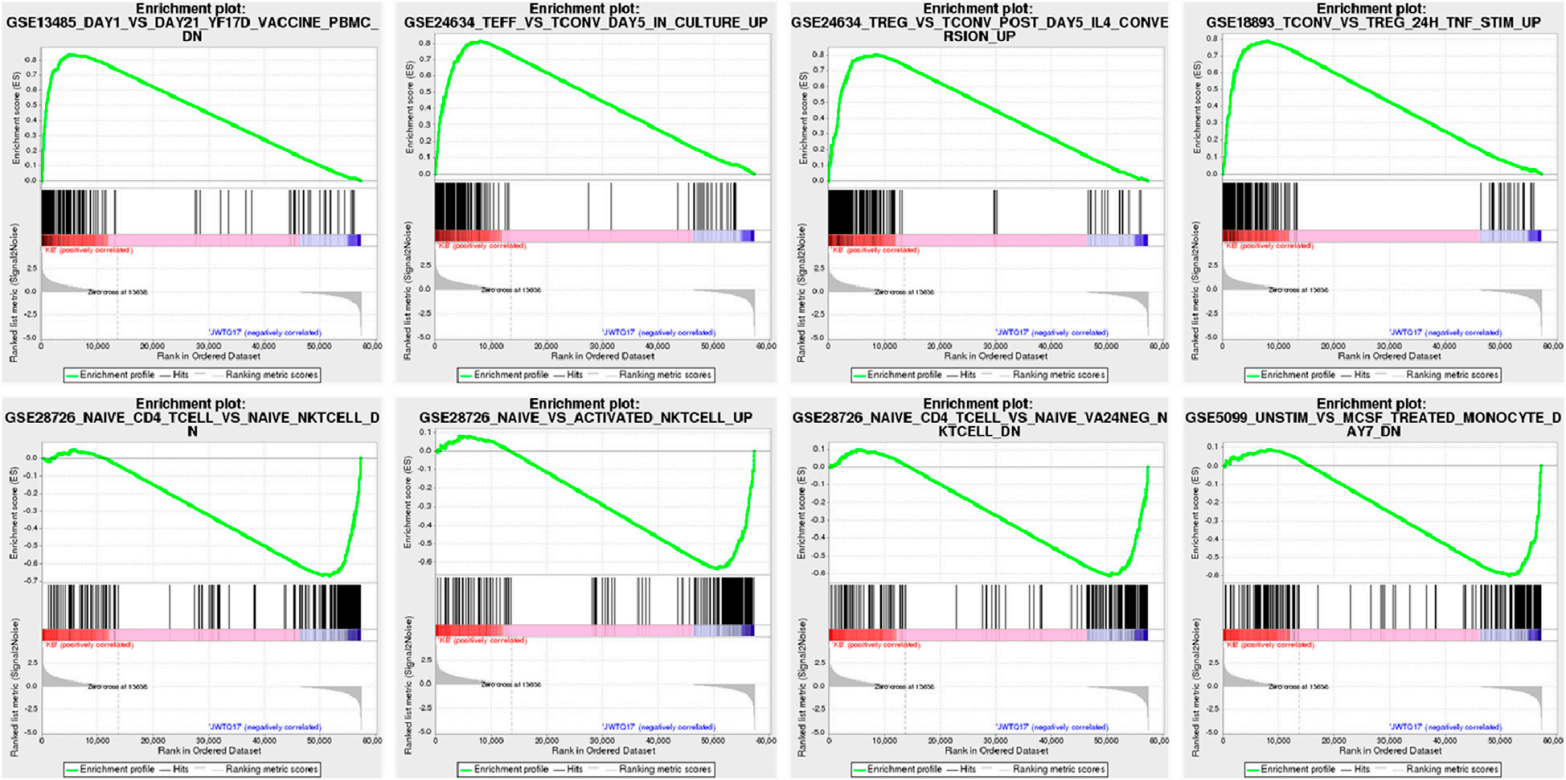
In the upper part of the graph, the dynamic ES values are represented, with the highest point indicating the ES value for this gene set. The middle part of the figure represents the sorted sequence of hybrid data, with vertical lines indicating genes present in this gene set in the sequenced dataset. The lower part of the curve represents the sorted values, distributed from high to low along the sequence of the sequenced data. In phenotype classification data files, positive values indicate correlation with the first phenotype, negative values indicate correlation with the second phenotype. In continuous phenotype data files, such as time series, positive values indicate positive correlation, negative values indicate negative correlation, or no correlation.

For the differential genes (results of differential expression analysis after metabolites intervention), GSEA KEGG analysis was performed using the WEBGestalt database (http://www.webgestalt.org), provided by Meiji Biosciences (Figure 13, Table 8).

**TABLE 8:** Results of GSEA enrichment analysis.

Gene set name	Description	ES	Padjust
GSE28726_NAIVE_CD4_TCELL_VS_NAIVE_NKTCELL_DN	Genes downregulated in naïve T cells: CD4 [GeneID = 920] *versus* NK.	−0.6719	0
GSE28726_NAIVE_VS_ACTIVATED_NKTCELL_UP	Genes upregulated in NKT cells: naïve *versus* activated	−0.63494	0
GSE28726_NAIVE_CD4_TCELL_VS_NAIVE_VA24NEG_NKTCELL_DN	Genes downregulated in naïve T cells: CD4 [GeneID = 920] *versus* Va24- NKT.	−0.61459	0.002661
GSE5099_UNSTIM_VS_MCSF_TREATED_MONOCYTE_DAY7_DN	Genes downregulated in unstimulated monocytes *versus* macrophages incubated with CSF1 [GeneID = 435] at day 7	−0.60051	0.00633
GSE18893_TCONV_VS_TREG_24H_TNF_STIM_UP	Genes upregulated in lymphocytes treated with TNF [GeneID = 7124] for 24H: T conv *versus* T reg cells	0.790745	0
GSE24634_TREG_VS_TCONV_POST_DAY5_IL4_CONVERSION_UP	Genes upregulated in comparison of CD25^+^ T cells treated with IL4 [GeneID = 3565] *versus* CD25^−^ T cells treated with IL4 [GeneID = 3565] at day 5	0.80377	0
GSE24634_TEFF_VS_TCONV_DAY5_IN_CULTURE_UP	Genes upregulated in comparison of untreated CD25^+^ T effector cells at day 5 *versus* untreated CD25^−^ T cells at day 5	0.815868	0
GSE13485_DAY1_VS_DAY21_YF17D_VACCINE_PBMC_DN	Genes downregulated in comparison of unstimulated peripheral blood mononuclear cells (PBMC) 1 day after stimulation with YF17D vaccine *versus* PBMC 21 days after the stimulation	0.839337	0

The six lowest (−0.67, −0.63, −0.61, −0.60, −0.60, −0.60) and highest (0.84, 0.82, 0.80, 0.79, 0.79, 0.77) standardized enrichment scores (ES) were all found to have P-values smaller than 0.01.

### 3.10 Transcription factor analysis

Transcription factors (TFs) are proteins that can bind to specific DNA sequences, widely found in organisms, and can activate or inhibit gene expression by binding to cis-regulatory elements upstream of genes. TFs usually consist of DNA-binding domains (DBDs), trans-activating domains (TADs), and other TF binding domains (Signal sensing domain, SSD). Based on different domains, TFs can be classified into different TF families, generally sharing the same DBD but different TADs.

By analyzing the domain information contained in gene transcripts, TF prediction and family analysis of genes were performed. JASPAR (http://jaspar.genereg.net/) and AnimalTFDB 3.0 (http://bioinfo.life.hust.edu.cn/AnimalTFDB/) were used to analyze TFs from human and murine genes, respectively. Additionally, TF-target gene prediction was performed to obtain potential regulatory gene information.

### 3.11 Expression distribution

Quantitative analysis was conducted on the overall expression levels of genes/transcripts using software, allowing for the subsequent analysis of differential gene expression among different samples. Boxplots and violin plots were used to illustrate the distribution of gene expression among different samples. Furthermore, by integrating sequence function information, gene regulatory mechanisms can be revealed (Supplementary Figure 2).

### 3.12 Predictive interaction network of key genes

**FIGURE 14:**
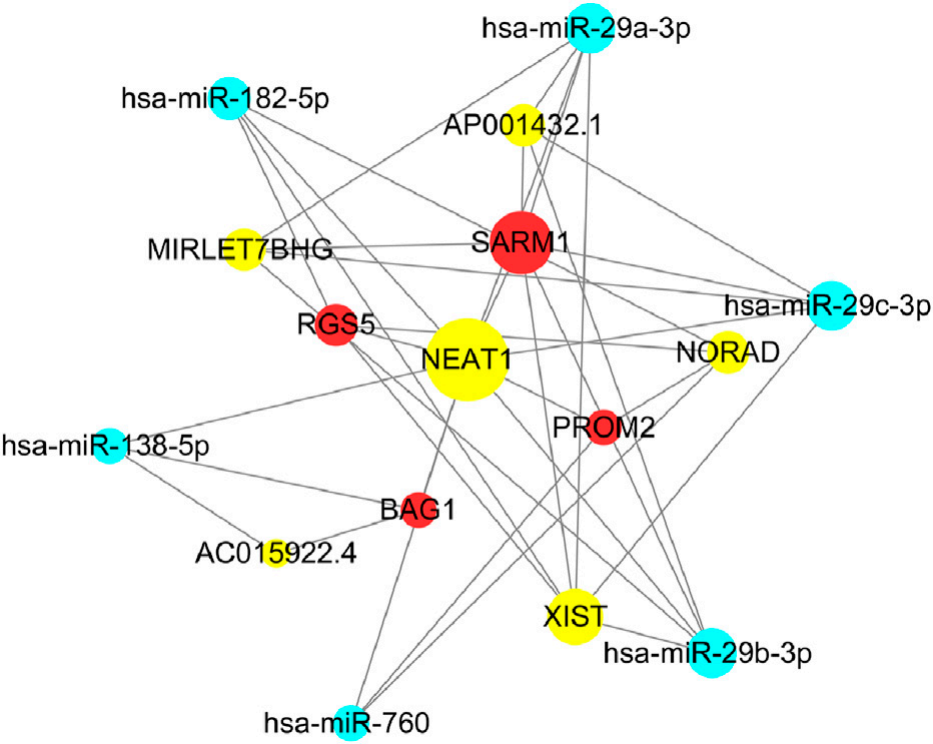
Clinical prognostic gene mRNA-lncRNA interaction network.

Using clinical prognosis genes (SARM1, RGS5, PROM2, BAG1), data from the miRNA-mRNA module were used to identify key miRNAs that highly co-express with mRNAs in breast cancer. Further, using data from the miRNA-lncRNA module, key miRNAs obtained were docked with lncRNA to identify lncRNAs interacting with key miRNAs, constructing an mRNA-lncRNA interaction network, as shown in Figure 14. We selected SARM1, RGS5, PROM2, BAG1, and NEAT1 targets in the network to further investigate their expression.

### 3.13 PCR experiment and western blot experimental results

During the preparation of cell samples, three different dosages were used to intervene with breast cancer cells: high-dose group (JH1/2/3), medium-dose group (JM1/2/3), and low-dose group (JL1/2/3). When high-concentration metabolitess were co-cultured with cells, a higher number of cells underwent apoptosis. Therefore, in the PCR results, the gene expression levels of SRAM1, PROM2, BAG1, and NEAT1 in the JH group were significantly lower compared to the other dosage groups. Hence, the medium-dose group (JM1/2/3) and low-dose group (JL1/2/3) hold greater reference value.

**FIGURE 15:**
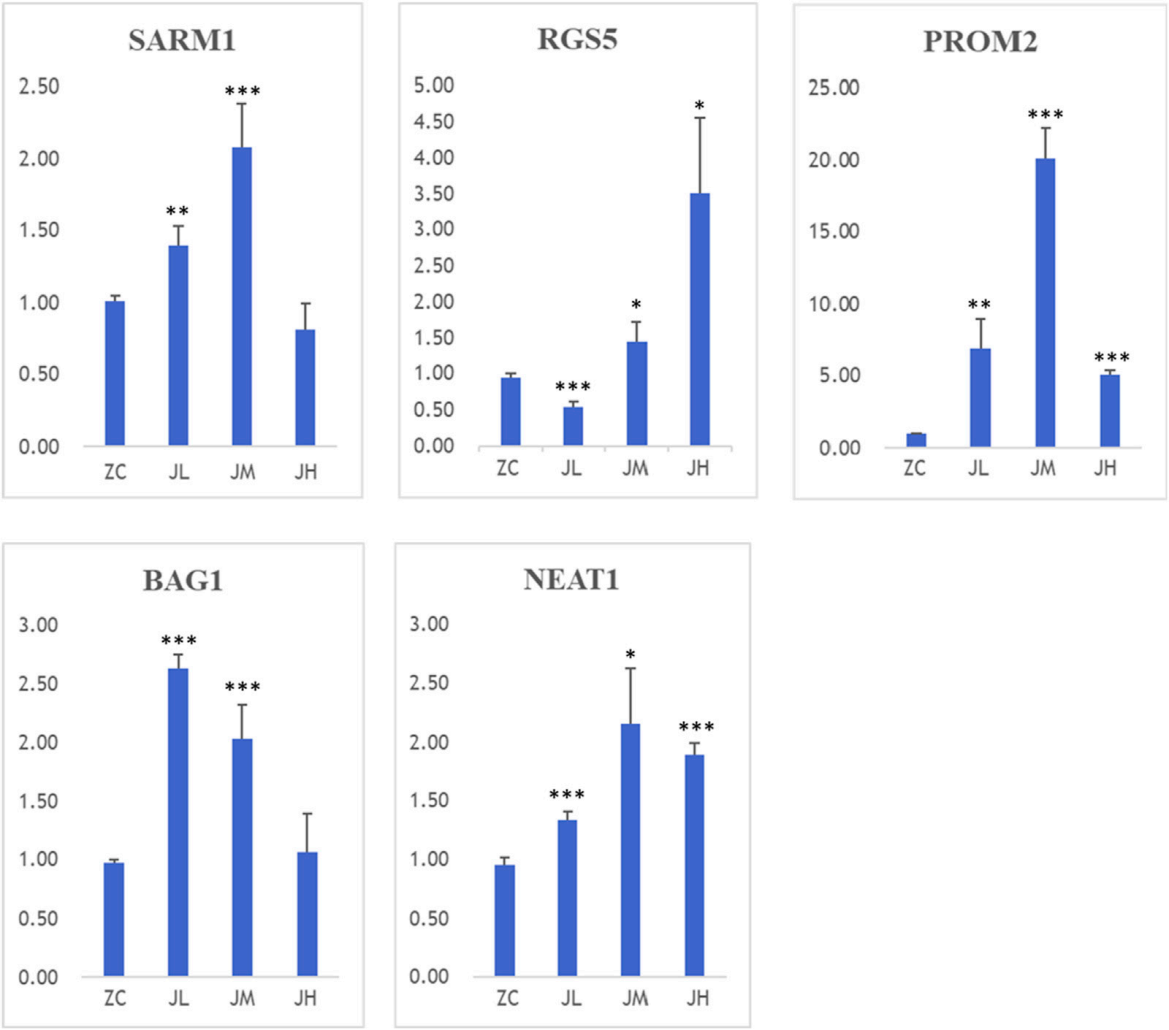
PCR results of SARM1, RGS5, BAG1, PROM2, NEAT1. ZC1/2/3:breast cancer normal control group, JL1/2/3: additive low dose group, JM1/2/3: additive heavy dose group, JH1/2/3 additive high dose group. Compared with blank group, *p < 0.05, **p < 0.01, ***p < 0.005.

The detection results revealed that, compared to the control group, both low and medium doses of metabolitess significantly upregulated the expression of SARM1, PROM2, BAG1, and NEAT1 genes. Moreover, the low-dose metabolitess exhibited a remarkable increase in the expression of the RGS5 gene (Figure 15).

**FIGURE 16:**
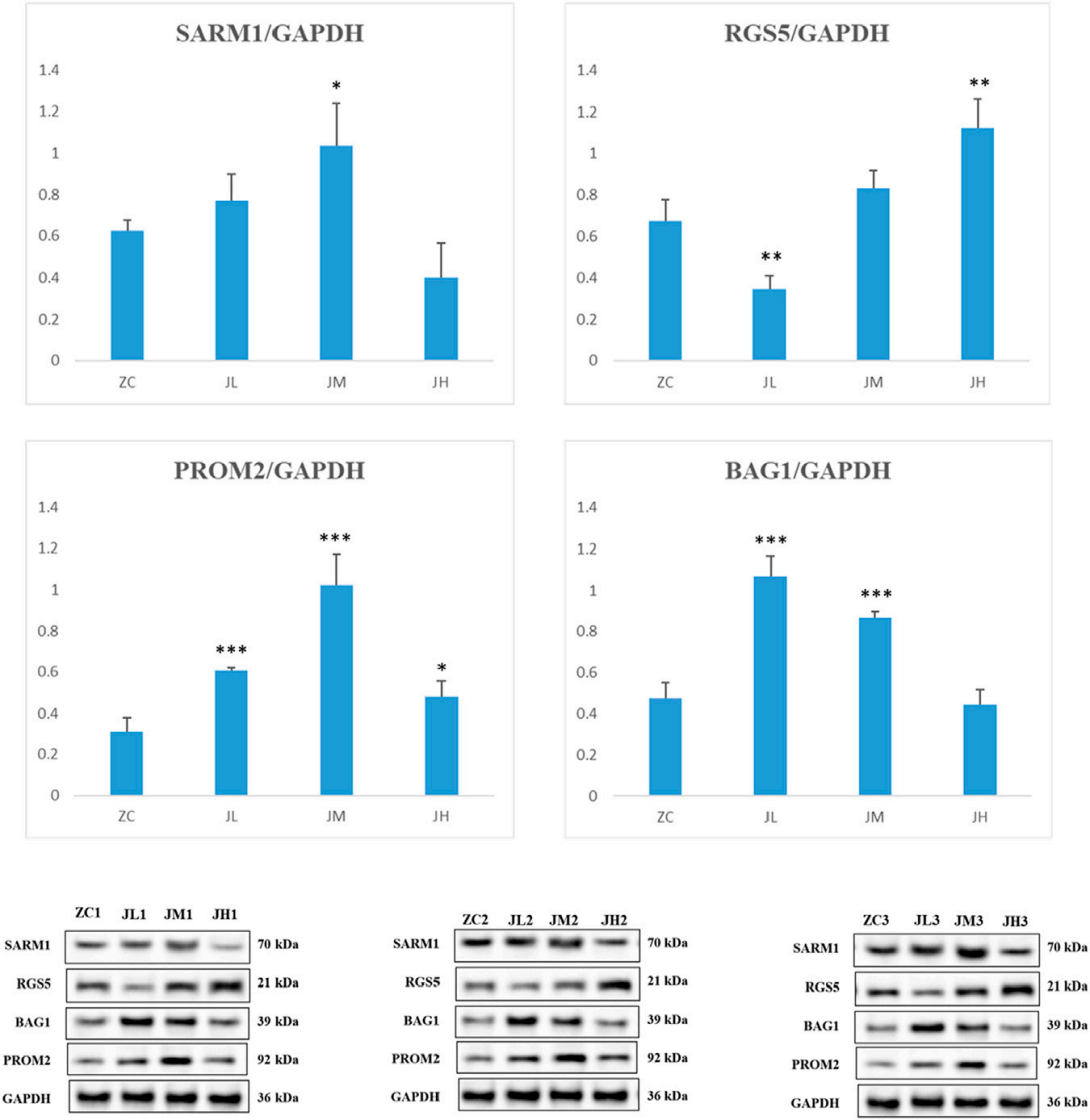
WB detection results of SARM1, RGS5, PROM2, BAG1. ZC1/2/3: breast cancer normal control group, JL1/2/3: additive low dose group, JM1/2/3: additive heavy dose group, JH1/2/3 additive high dose group. Compared with blank group, *p < 0.05, **p < 0.01, ***p < 0.005.

The results of the WB (Western blot) experiments were similar to those of the PCR experiments. When compared with the blank control group, the metabolites treatment significantly increased the protein levels of SARM1, PROM2, and BAG1, while decreasing the protein expression levels of RGS5 (Figure 16).

### 3.14 Determination of metabolites hemocompatibility

*Hemolysis Rate* The experimental results demonstrated that the hemolysis rate in the high-concentration group (H group) was significantly higher than that in the medium-concentration (M group) and low-concentration groups (L group) (p < 0.05). This suggests that the metabolites at high concentrations may cause significant damage to the erythrocyte membrane, posing a potential risk of hemolytic toxicity.

*Coagulation Function Assessment* Prothrombin Time (PT), Thrombin Time (TT), Activated Partial Thromboplastin Time (APTT), and Fibrinogen (Fib) Content: No significant changes were observed across all groups (p > 0.05), indicating that the metabolites did not exert a noticeable inhibitory effect on thrombin generation or coagulation pathways at any concentration.

*Plasma Protein Measurement* Albumin (Alb): Compared to the control group (NC group), albumin levels in the high-concentration and medium-concentration groups were significantly reduced (p < 0.05), suggesting that the metabolites may impair albumin synthesis in the liver or increase its consumption. Total Protein (TP): The total protein level in the medium-concentration group was significantly higher than in other groups, indicating that the metabolites might induce an inflammatory response or immune activation, leading to an increase in proteins such as immunoglobulins. Immunoglobulin G (IgG): The IgG level in the high-concentration group was significantly lower than that in the control group (p < 0.05), suggesting a potential immunosuppressive effect of the metabolites. Fibrinogen (Fbg): The fibrinogen levels in the high-concentration and low-concentration groups were significantly lower than those in the control group, indicating that the metabolites may interfere with fibrinogen metabolism, potentially impacting blood coagulation.

*Dynamic Coagulation Time* The dynamic coagulation time in all groups showed a gradual decline over time, indicating that the metabolites did not significantly interfere with coagulation function. However, slightly higher absorbance values in the high-concentration group may suggest a mild anticoagulant effect.

The results indicate that the metabolites exhibits dose-dependent effects on blood compatibility: The high-concentration group demonstrated a higher hemolysis rate and lower levels of immunoglobulin G and fibrinogen, suggesting potential hemolytic toxicity and immunosuppressive effects. The elevated total protein levels in the medium-concentration group may reflect an enhanced inflammatory response or immune activation. The low-concentration group showed minor effects on fibrinogen metabolism.

**FIGURE 17:**
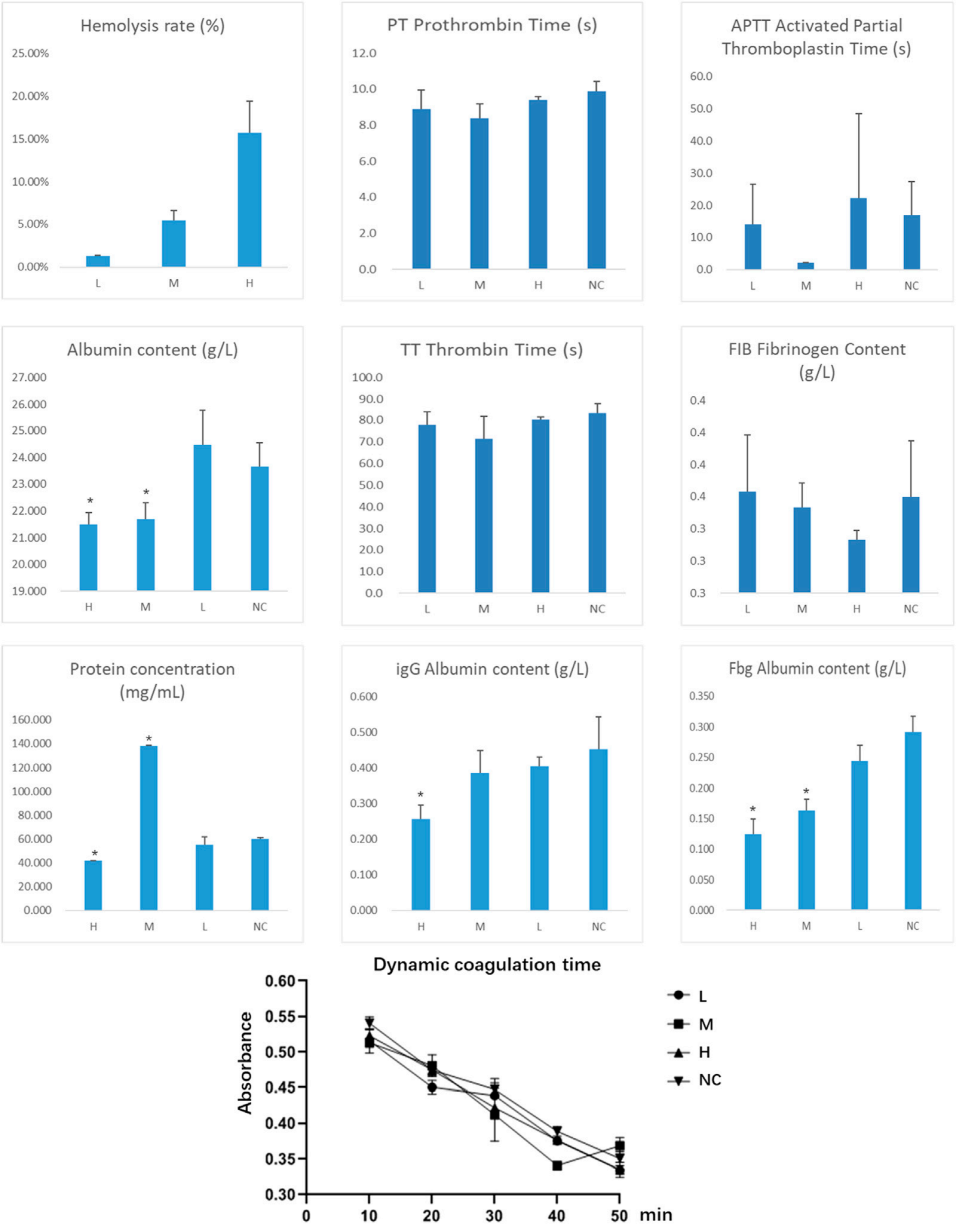
Determination of Metabolites Hemocompatibility (Indicates a significant difference compared to the normal control group (NC), with *p* < 0.05).

This study utilized various metabolites doses. During the preliminary experiments, it was found that the concentrated dose of fungal extract was too high for cellular experiments, yielding no viable results. After dilution and combining the findings from the cellular experiments, it is recommended that future *in vivo* animal studies use the low-dose concentration determined from the hemolysis assay to minimize potential risks. During the dose design and application process, it is essential to carefully consider the potential risks to hemocompatibility and control the metabolites concentration to ensure safety (Figure 17).

With the present study, we determined the key role of the metabolites of the endophytic bacterium *P. frederiksbergensis* in the suppression of breast cancer cells. We found that which are associated with key pathways influencing tumor progression, immune modulation, and cellular apoptosis. The constructed clinical oncogenic action network provided us with a deeper understanding of the mechanism of action of endophyte metabolites on breast cancer cells. In addition, the validation by PCR and WB experiments further established the exact regulatory effects of endophyte metabolites on these targets. These findings not only expand our understanding of endophytic bacterial metabolites, but also provide valuable new ideas and potential clinical applications for breast cancer treatment and prognosis.

Among these, genes such as *SARM1, RGS5, PROM2, and BAG1* have emerged as critical players in breast cancer biology, influencing cell survival, migration, and resistance to treatment. ***SARM1*** *(Sterile Alpha and TIR Motif-Containing 1)* plays a role in regulating cell death pathways and immune responses, which are pivotal in cancer progression (Butt et al., 2008)*.* ***RGS5*** *(Regulator of G-protein signaling 5*) is associated with tumor angiogenesis and resistance to chemotherapy (Yin et al., 2023)*.* ***PROM2*** *(Prominin 2) is involved in stem cell* maintenance and has been linked to cancer stem cell properties in breast cancer (Piezzo et al., 2020)*.* ***BAG1*** *(Bcl-2-associated athanogene 1)* modulates protein stability and apoptosis, influencing chemotherapy resistance in breast cancer cells (Yin et al., 2023).

These genes were chosen for investigation due to their critical roles in cancer cell survival, proliferation, and resistance mechanisms. The metabolites of *P. frederiksbergensis* may offer a novel approach to modulating these pathways, providing an alternative therapeutic avenue with potentially fewer side effects compared to current treatments.

Currently, the standard therapies for breast cancer—chemotherapy, surgery, and radiation—often lead to significant side effects and are limited by resistance, particularly in metastatic cases. *Targeted* therapies, such as CDK4/6 inhibitors, have shown promise in overcoming resistance, but their effectiveness can be limited by the heterogeneity of tumors and the development of new resistance mechanisms (Piezzo et al., 2020)*.* In contrast, the metabolites from *P. frederiksbergensis* represent a promising alternative due to their ability to influence multiple signaling pathways, including apoptosis and cell cycle regulation, in a more targeted and potentially less toxic manner.

Further investigations revealed that the secondary metabolites from *P. frederiksbergensis* might exert their effects on breast cancer cells by influencing specific signaling pathways, including cell cycle regulation, apoptotic processes, and cell proliferation. This discovery opens new avenues for targeted therapies in breast cancer treatment. For instance, the modulation of genes such as SARM1, RGS5, PROM2, and BAG1 could be directly linked to tumor cell survivability, invasiveness, and metabolites sensitivity. Moreover, a deeper analysis of how these metabolites affect key points in the cell cycle could elucidate their potential mechanisms in inhibiting tumor growth. Lastly, the bioinformatics analysis conducted in this study also sheds light on how these metabolites might alter the microenvironment of breast cancer cells by affecting gene expression and signaling networks, guiding future experimental designs and clinical research directions.

Additionally, our study suggests that the anti-cancer properties of these metabolites may involve the modulation of immune responses, particularly in the tumor microenvironment. The interaction between these metabolites and key immune cells, such as T-cells and macrophages, could be critical in mediating anti-tumor effects. This highlights the potential of these secondary metabolites as immunomodulatory agents in breast cancer therapy (Xie and Chen, 2023).

Furthermore, the potential synergistic effects of these metabolites with existing chemotherapy agents should be explored. Combining these natural metabolites with established treatments could enhance efficacy and reduce side effects, offering a more holistic approach to cancer management.

In light of these findings, it is imperative to conduct *in vivo* studies to validate these *in vitro* results and to understand the pharmacokinetics and pharmacodynamics of these metabolites in animal models. Such studies will provide crucial insights into the feasibility and safety of using these metabolites in clinical settings.

To fully harness the therapeutic potential of these metabolites, future research should also focus on their chemical characterization, stability, and bioavailability. Understanding these aspects will be key to developing efficient metabolites delivery systems and formulating these metabolites into viable therapeutic agents.

In conclusion, the secondary metabolites of *P. frederiksbergensis* offer promising avenues for the development of novel anti-cancer therapies. Their diverse mechanisms of action, coupled with the potential for low toxicity, make them valuable candidates for further research and clinical exploration.

## 4 Conclusion

The metabolites of endophytic bacterium *P. frederiksbergensis* from Taoerqi exhibit cytotoxicity against breast cancer. The clinical prognosis risk-score model provides insights into risk stratification and potential molecular pathways associated with breast cancer progression. Based on bioinformatics analysis, the metabolites of *P. frederiksbergensis* can enhance the survival period of breast cancer patients. These metabolites can act through intervening in breast cancer targets such as SARM1, RGS5, BAG1, PROM2, and NEAT1.

## Data Availability

The original contributions presented in the study are publicly available. This data can be found here: https://www.ncbi.nlm.nih.gov/, PRJNA1242223.
