# Supplementary material for: Integrative analysis of anti-breast CancerPotential of metabolites from Pseudomonas frederiksbergensis isolated from Taoerqi
Source: Front Pharmacol. 2025 Mar 28;16:1469949. doi: 10.3389/fphar.2025.1469949 (PMC11987712; doi:10.3389/fphar.2025.1469949)
Supplement: Supplementary file 1 [file Supplementaryfile1.docx]

***Supplementary Material***

1. **Supplementary Data**

GCATGGCGCGGGCTACACATGCAGTCGAGCGGCAGCACGGGTACTTGTACCTGGTGGCGAGCGGCGGACGGGTGAGTAATGCCTAGGAATCTGCCTGGTAGTGGGGGATAACGCTCGGAAACGGACGCTAATACCGCATACGTCCTACGGGAGAAAGCAGGGGACCTTCGGGCCTTGCGCTATCAGATGAGCCTAGGTCGGATTAGCTAGTTGGTGAGGTAATGGCTCACCAAGGCGACGATCCGTAACTGGTCTGAGAGGATGATCAGTCACACTGGAACTGAGACACGGTCCAGACTCCTACGGGAGGCAGCAGTGGGGAATATTGGACAATGGGCGAAAGCCTGATCCAGCCATGCCGCGTGTGTGAAGAAGGTCTTCGGATTGTAAAGCACTTTAAGTTGGGAGGAAGGGCATTTACCTAATACGTAAGTGTTTTGACGTTACCGACAGAATAAGCACCGGCTAACTCTGTGCCAGCAGCCGCGGTAATACAGAGGGTGCAAGCGTTAATCGGAATTACTGGGCGTAAAGCGCGCGTAGGTGGTTTGTTAAGTTGGATGTGAAATCCCCGGGCTCAACCTGGGAACTGCATTCAAAACTGACAAGCTAGAGTATGGTAGAGGGTGGTGGAATTTCCTGTGTAGCGGTGAAATGCGTAGATATAGGAAGGAACACCAGTGGCGAAGGCGACCACCTGGACTGATACTGACACTGAGGTGCGAAAGCGTGGGGAGCAAACAGGATTAGATACCCTGGTAGTCCACGCCGTAAACGATGTCAACTAGCCGTTGGGAGCCTTGAGCTCTTAGTGGCGCAGCTAACGCATTAAGTTGACCGCCTGGGGAGTACGGCCGCAAGGTTAAAACTCAAATGAATTGACGGGGGCCCGCACAAGCGGTGGAGCATGTGGTTTAATTCGAAGCAACGCGAAGAACCTTACCAGGCCTTGACATCCAATGAACTTTCCAGAGATGGATTGGTGCCTTCGGGAACATTGAGACAGGTGCTGCATGGCTGTCGTCAGCTCGTGTCGTGAGATGTTGGGTTAAGTCCCGTAACGAGCGCAACCCTTGTCCTTAGTTACCAGCACGTAATGGTGGGCACTCTAAGGAGACTGCCGGTGACAAACCGGAGGAAGGTGGGGATGACGTCAAGTCATCATGGCCCTTACGGCCTGGGCTACACACGTGCTACAATGGTCGGTACAGAGGGTTGCCAAGCCGCGAGGTGGAGCTAATCCCAGAAAACCGATCGTAGTCCGGATCGCAGTCTGCAACTCGACTGCGTGAAGTCGGAATCGCTAGTAATCGCGAATCAGAATGTCGCGGTGAATACGTTCCCGGGCCTTGTACACACCGCCCGTCACACCATGGGAGTGGGTTGCACCAGAAGTAGCTAGTCTAACCTTCGGGAGGACGGTACCACGGTGATAA

>G(17)

CGGGCGGGGGCCCTAACACATGCAGTCGAGCGGCAGCACGGGTACTTGTACCTGGTGGCGAGCGGCGGACGGGTGAGTAATGCCTAGGAATCTGCCTGGTAGTGGGGGATAACGCTCGGAAACGGACGCTAATACCGCATACGTCCTACGGGAGAAAGCAGGGGACCTTCGGGCCTTGCGCTATCAGATGAGCCTAGGTCGGATTAGCTAGTTGGTGAGGTAATGGCTCACCAAGGCGACGATCCGTAACTGGTCTGAGAGGATGATCAGTCACACTGGAACTGAGACACGGTCCAGACTCCTACGGGAGGCAGCAGTGGGGAATATTGGACAATGGGCGAAAGCCTGATCCAGCCATGCCGCGTGTGTGAAGAAGGTCTTCGGATTGTAAAGCACTTTAAGTTGGGAGGAAGGGCATTTACCTAATACGTAAGTGTTTTGACGTTACCGACAGAATAAGCACCGGCTAACTCTGTGCCAGCAGCCGCGGTAATACAGAGGGTGCAAGCGTTAATCGGAATTACTGGGCGTAAAGCGCGCGTAGGTGGTTTGTTAAGTTGGATGTGAAATCCCCGGGCTCAACCTGGGAACTGCATTCAAAACTGACAAGCTAGAGTATGGTAGAGGGTGGTGGAATTTCCTGTGTAGCGGTGAAATGCGTAGATATAGGAAGGAACACCAGTGGCGAAGGCGACCACCTGGACTGATACTGACACTGAGGTGCGAAAGCGTGGGGAGCAAACAGGATTAGATACCCTGGTAGTCCACGCCGTAAACGATGTCAACTAGCCGTTGGGAGCCTTGAGCTCTTAGTGGCGCAGCTAACGCATTAAGTTGACCGCCTGGGGAGTACGGCCGCAAGGTTAAAACTCAAATGAATTGACGGGGGCCCGCACAAGCGGTGGAGCATGTGGTTTAATTCGAAGCAACGCGAAGAACCTTACCAGGCCTTGACATCCAATGAACTTTCCAGAGATGGATTGGTGCCTTCGGGAACATTGAGACAGGTGCTGCATGGCTGTCGTCAGCTCGTGTCGTGAGATGTTGGGTTAAGTCCCGTAACGAGCGCAACCCTTGTCCTTAGTTACCAGCACGTAATGGTGGGCACTCTAAGGAGACTGCCGGTGACAAACCGGAGGAAGGTGGGGATGACGTCAAGTCATCATGGCCCTTACGGCCTGGGCTACACACGTGCTACAATGGTCGGTACAGAGGGTTGCCAAGCCGCGAGGTGGAGCTAATCCCAGAAAACCGATCGTAGTCCGGATCGCAGTCTGCAACTCGACTGCGTGAAGTCGGAATCGCTAGTAATCGCGAATCAGAATGTCGCGGTGAATACGTTCCCGGGCCTTGTACACACCGCCCGTCACACCATGGGAGTGGGTTGCACCAGAAGTAGCTAGTCTAACCTTCGGGAGGACGGTACCACGGTGATTACGC

1. **Supplementary Figures and Tables**

**
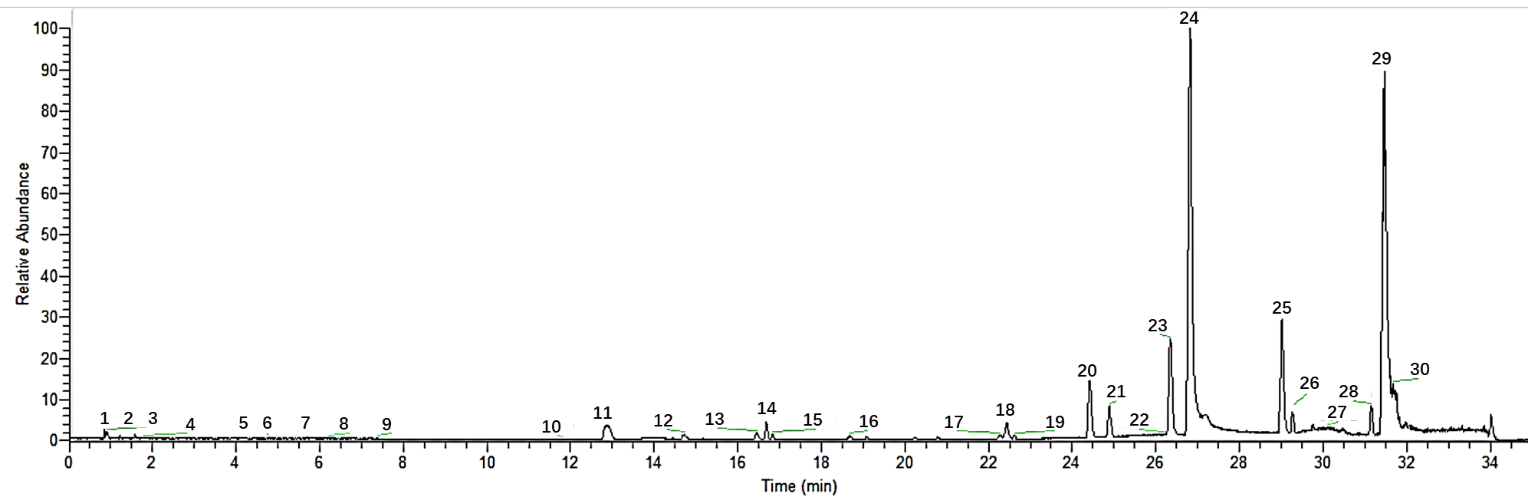
**

**Supplementary Figure 1.** Chemical fingerprint details of *Pseudomonas frederiksbergensis* metabolites extract samples (detected using Thermo Fisher ABScience LC-MS)

**Supplementary Table 1**  Chemical fingerprint details of *Pseudomonas frederiksbergensis* metabolites extract samples

| No. | Time  （min） | Measured  （m/z） | Theoretical  （m/z） | Error  （ppm） | Theoretical Formula |
| --- | --- | --- | --- | --- | --- |
| 1 | 0.85 | 239.0128 | 238.0218 | 0.02 | C_17_H_3_O_2_ |
| 1 | 0.85 | 301.1396 | 301.1394 | 0.49 | C_13_H_21_O_6_N_2_ |
| 2 | 1.23 | 158.0914 | 158.0911 | 1.83 | C_4_H_10_ON_6_ |
| 3 | 1.58 | 155.0805 | 155.0802 | 2.51 | C_5_H_9_ON_5_ |
| 4 | 1.78 | 149.0223 | 149.0220 | 2.5 | C_6_H_3_O_2_N_3_ |
| 5 | 4.22 | 279.1574 | 279.1577 | -1.19 | C_14_H_21_O_3_N_3_ |
| 6 | 4.79 | 553.2870 | 553.2881 | -0.87 | C_27_H_37_O_5_N_8_ |
| 7 | 5.70 | 227.1378 | 227.1377 | 0.5 | C_9_H_17_O_2_N_5_ |
| 8 | 6.21 | 261.1216 | 261.1220 | -0.87 | C_12_H_15_O_2_N_5_ |
| 9 | 7.38 | 539.3153 | 539.3146 | 1.04 | C_20_H_43_O_9_N_8_ |
| 10 | 11.80 | 229.0492 | 229.0495 | -1.38 | C_13_H_9_O_4_ |
| 11 | 12.84 | 205.1423 | 205.1421 | 1.25 | C_8_H_19_O_3_N_3_ |
| 11 | 12.84 | 227.1241 | 227.1238 | 1.53 | C_7_H_19_O_6_N_2_ |
| 12 | 14.72 | 158.1529 | 158.1526 | 2.04 | C_7_H_18_N_4_ |
| 13 | 16.64 | 284.1065 | 284.1062 | 1.04 | C_4_H_14_O_6_N_9_ |
| 14 | 16.69 | 274.2725 | 274.2727 | -0.7 | C_14_H_34_ON_4_ |
| 15 | 16.83 | 318.2984 | 318.2989 | -1.56 | C_16_H_38_O_2_N_4_ |
| 16 | 18.68 | 170.0954 | 170.0964 | -5.97 | C_12_H_12_N |
| 17 | 22.28 | 279.1574 | 279.1577 | -1.3 | C_14_H_21_O_3_N_3_ |
| 18 | 22.43 | 205.0848 | 205.0846 | 1.13 | C_10_H_11_O_2_N_3_ |
| 19 | 22.62 | 212.1421 | 212.1434 | -5.78 | C_15_H_18_N |
| 20 | 24.43 | 403.2302 | 403.2300 | 0.97 | C_16_H_31_O_6_N_6_ |
| 21 | 24.89 | 280.2617 | 280.2621 | -1.53 | C_16_H_32_N_4_ |
| 21 | 26.19 | 338.3397 | 338.3404 | -2.01 | C_20_H_42_N_4_ |
| 22 | 26.34 | 256.2620 | 256.2621 | -0.74 | C_14_H_32_N_4_ |
| 23 | 26.81 | 282.2771 | 282.2778 | -2.58 | C_16_H_34_N_4_ |
| 23 | 29.02 | 284.2930 | 284.2934 | -1.47 | C_16_H_36_N_4_ |
| 24 | 29.28 | 310.3086 | 310.3091 | -1.64 | C_18_H_38_N_4_ |
| 25 | 29.75 | 336.3242 | 336.3253 | -3.38 | C_20_H_40_N_4_ |
| 26 | 31.15 | 461.3268 | 461.3267 | 0.24 | C_28_ H_45_O_5_ |
| 27 | 31.46 | 338.3392 | 338.3409 | -5.16 | C_20_H_42_N_4_ |

**\****Supplementary Table 2.** Cell inhibition rate of *Pseudomonas frederiksbergensis* fermentation extracts (two replicates)

| Group | A490 | | | Inhibition rate | | | | |
| --- | --- | --- | --- | --- | --- | --- | --- | --- |
|  | **24H** | **48H** | **72H** | **24H** | | **48H** | | **72H** |
| Medium+cells | 2.0808±0.2048 | 2.0601±0.4039 | 2.8169±0.1679 |  |  | |  | |
| Medium | 0.05 | 0.05 | 0.05 |  | |  | |  |
| Medium+metabolites | 0.1051±0.0163 | 0.2329±0.0026 | 0.9666±0.0695 |  | |  | |  |
| Medium+metabolites+cells | 1.3529±0.0819^**^ | 1.3284±0.1670^*^ | 2.3892±0.1892^*^ | 38.56% | | 45.50% | | 48.59% |

Compared with Medium + cells *P＜0.05，**P＜0.01


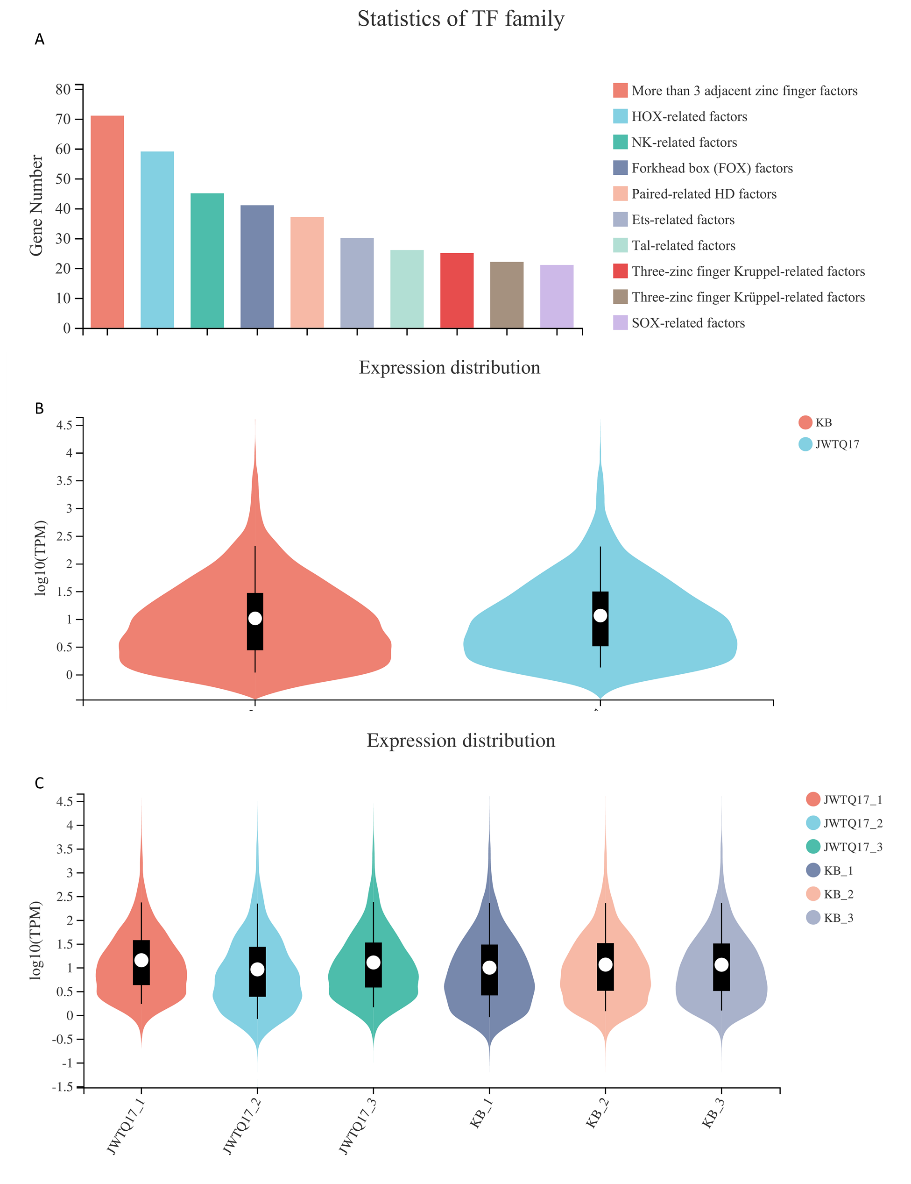


**Supplementary Figure 2.** A. Transcription factor analysis, with the x-axis indicating different transcription factor families and the y-axis indicating the number of genes falling into that transcription factor family. Genes are shown in ascending order of E value (-log10); on the right are the transcription factor families. B. Distribution of gene expression levels in the sequencing data quality control—Blank group (KB) versus metabolites-administered group (JWTQ17). C. Distribution of gene expression levels in the sequencing data quality control—Blank group (KB1, KB2, KB3) versus metabolites-administered group (JWTQ17-1, JWTQ17-2, JWTQ17-3) (x-axis represents the sample names/group names in this project; y-axis represents the logarithm to the base 10 of the expression level. Each color in the graph represents a sample/group; the swollen part of the graph represents the most concentrated area of gene expression in the entire sample).
